# Monocyte-related markers as predictors of immune checkpoint inhibitor efficacy and immune-related adverse events: a systematic review and meta-analysis

**DOI:** 10.1007/s10555-025-10246-6

**Published:** 2025-02-21

**Authors:** Aiarpi Ezdoglian, Michel Tsang-A-Sjoe, Fatemeh Khodadust, George Burchell, Gerrit Jansen, Tanja de Gruijl, Mariette Labots, Conny J. van der Laken

**Affiliations:** 1 Department of Rheumatology and Clinical Immunology, Amsterdam University Medical Center, Amsterdam, The Netherlands; 2 Amsterdam University Medical Library, Amsterdam, The Netherlands; 3 Department of Medical Oncology, Cancer Center Amsterdam, Amsterdam University Medical Center, Location Vrije Universiteit, Amsterdam, The Netherlands; 4 Department of Medical Oncology, Amsterdam University Medical Center, Location Vrije Universiteit, Amsterdam, The Netherlands

**Keywords:** Monocytes, Immune checkpoint inhibitors, Immune-related adverse events, M-MDSCs, Monocytic myeloid-derived suppressor cells, PD-1, PD-L1, CTLA-4

## Abstract

The efficacy and off-target effects of immune checkpoint inhibitors (ICI) in cancer treatment vary among patients. Monocytes likely contribute to this heterogeneous response due to their crucial role in immune homeostasis. We conducted a systematic review and meta-analysis to evaluate the impact of monocytes on ICI efficacy and immune-related adverse events (irAEs) in patients with cancer. We systematically searched PubMed, Web of Science, and Embase for clinical studies from January 2000 to December 2023. Articles were included if they mentioned cancer, ICI, monocytes, or any monocyte-related terminology. Animal studies and studies where ICIs were combined with other biologics were excluded, except for studies where two ICIs were used. This systematic review was registered with PROSPERO (CRD42023396297) prior to data extraction and analysis. Monocyte-related markers, such as absolute monocyte count (AMC), monocyte/lymphocyte ratio (MLR), specific monocyte subpopulations, and m-MDSCs were assessed in relation to ICI efficacy and safety. Bayesian meta-analysis was conducted for AMC and MLR. The risk of bias assessment was done using the Cochrane-ROBINS-I tool. Out of 5787 studies identified in our search, 155 eligible studies report peripheral blood monocyte-related markers as predictors of response to ICI, and 32 of these studies describe irAEs. Overall, based on 63 studies, a high MLR was a prognostic biomarker for short progression-free survival (PFS) and overall survival (OS) hazard ratio (HR): 1.5 (95% CI: 1.21–1.88) and 1.52 (95% CI:1.13–2.08), respectively. The increased percentage of classical monocytes was an unfavorable predictor of survival, while low baseline rates of monocytic myeloid-derived suppressor cells (m-MDSCs) were favorable. Elevated intermediate monocyte frequencies were associated but not significantly correlated with the development of irAEs. Baseline monocyte phenotyping may serve as a composite biomarker of response to ICI; however, more data is needed regarding irAEs. Monocyte-related variables may aid in risk assessment and treatment decision strategies for patients receiving ICI in terms of both efficacy and safety.

**Supplementary Information:**

The online version contains supplementary material available at 10.1007/s10555-025-10246-6.

## Introduction

The introduction of immune checkpoint inhibitors (ICIs) in cancer immunotherapy has revolutionized treatment, offering new hope for many patients [1]. With the increasing number of clinically available ICIs and their combinations, new insights into efficacy and safety are being uncovered [2, 3]. While most ICI research has been T cell-centered, myeloid cells, including monocytes, macrophages, and monocytic myeloid-derived suppressor cells (mDSCs), have recently gained attention due to their emerging role in both the efficacy and the toxicity of ICI therapy [4, 5].

Currently approved ICIs target CTLA-4, PD-1/PD-L1, or LAG-3 pathways, with most (pre-)clinical experience accumulated for the first two. CTLA-4 is highly expressed in immune-suppressive regulatory T cells (Tregs) and dampens the activation of helper and cytotoxic T lymphocytes [6]. Reducing the number of CTLA-4-positive T-regs is a vital mechanism underlying the antitumor efficacy of anti-CTLA-4 antibodies. PD-1, binding to its ligand PD-L1, blocks the activation of immune effector cells, thus promoting and maintaining anti-inflammatory conditions [7]. In the tumor microenvironment, PD-1 engagement leads to T-cell exhaustion [8]. Anti-PD-L1 therapy helps restore immune function by blocking this interaction. Anti–PD-L1 therapy promotes a direct and distinct inflammatory signature in CD14^+^ monocytes and increases myeloid-derived cytokines [9].

The safety and efficacy of ICIs vary among patients, as well as across different immunotherapy and tumor types [10]. In several indications, ICIs are combined, enhancing their efficacy and increasing their toxicity [11]. In healthy tissues, checkpoint molecules maintain homeostasis and prevent autoimmunity. ICIs disrupt this equilibrium of auto-tolerance and can provoke an array of immune-related adverse events (irAEs), including endocrine, gastrointestinal, and rheumatic complications [12].

Systemic biomarkers help to monitor treated patients over time, reducing reliance on inconsistent tumor biopsies. Predictors of the response to ICI can be divided into tumor-derived and host-immunity-derived parameters and can used solely or in combination [13]. Immunosuppressive monocyte phenotypes have been implicated in various diseases, including cancer. Monocyte subpopulations, namely classical, non-classical, and intermediate, differ primarily in their phenotypic characteristics and functional roles within the immune system. Classical monocytes can eliminate cancer cells through direct phagocytosis. On the other hand, they become prooncogenic tumor-associated macrophages [14]. Non-classical monocytes eliminate cancer cells and can also prevent metastasis, while intermediate monocytes have the potency to become either classical or non-classical monocytes [15–17].

Certain monocyte phenotypes are associated with resistance to ICI therapy, while others correlate with a better response [4]. Among myeloid cells, monocytes and monocyte-derived macrophages play a critical role in the immune response and have been linked to several autoimmune diseases, such as rheumatoid arthritis, asthma, and Crohn’s disease [18]. Recent research also suggests monocyte involvement in the onset of irAEs [19, 20]. Thus, defining their exact contribution to ICI-therapy response and irAEs will be crucial for the optimal use of ICIs and minimizing potential adverse events. This systematic review investigates the relationship between baseline monocyte-related variables, ICI efficacy, and irAE development.

## Methods

### Search strategy and selection criteria

**Fig. 1:**
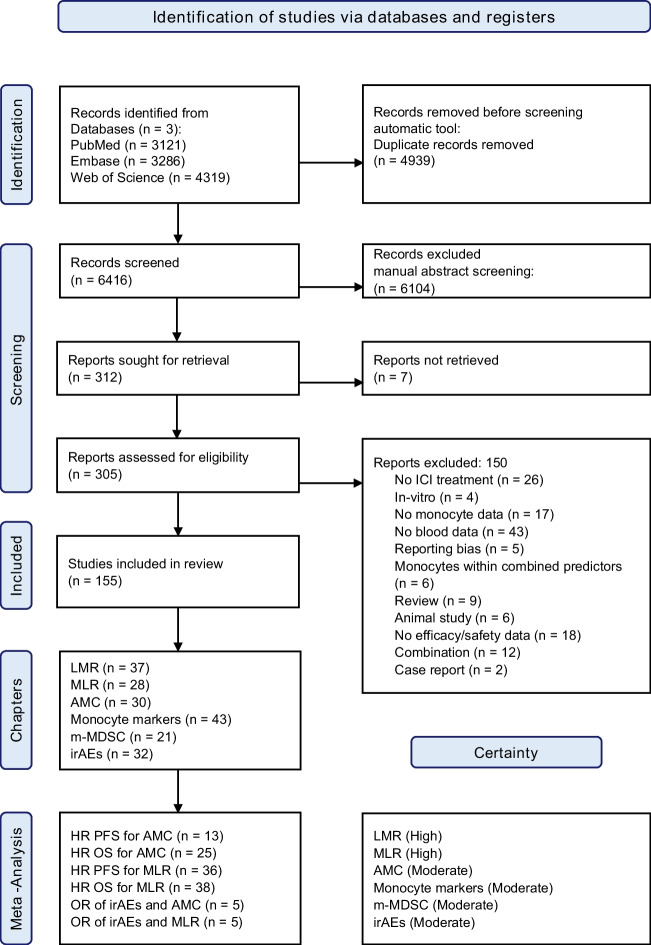
PRISMA flow diagram for study selection

Three databases—PubMed, Embase, and Web of Science—were searched for research articles published between January 2000 and December 2023, as ICI had not been reported for human use before 2000. Only human clinical studies were included, regardless of the language. This systematic review was registered with PROSPERO (registration no. CRD42023396297) prior to data extraction and analysis. The current systematic review adhered to the Preferred Reporting Items for Systematic Reviews and Meta-Analysis (PRISMA) statement (Fig. 1).

The search was performed using a combination of all clinically applied ICIs (in the pre-specified time frame), solid and hematological tumor types, and monocytes (Appendix 1.1). All titles/abstracts identified in the electronic databases were independently screened for eligibility criteria by two authors (AE, MT). Discrepancies were resolved through discussion; if consensus could not be reached, a third author (FK) provided additional screening to finalize inclusion.

Articles were included if they mentioned cancer, ICI, monocytes, or any monocyte-related terminology. Animal studies were manually excluded. The search strategy and selection criteria are detailed in Appendix 1.1–1.3. All potentially relevant full texts were screened by three authors (AE, MT, and FK). Initially, AE screened all articles and extracted the data, whereas MT and FK independently screened the same articles for extraction errors. Discrepancies were resolved through discussion. Indications, number of patients, presence of control groups, monocyte markers, monocyte-related cytokines, monocyte/lymphocyte ratio (MLR), lymphocyte/monocyte ratios (LMR), and the reporting of irAEs were extracted (Appendix 1.4–1.7). All articles included were subdivided into categories according to the study’s focus. Articles covering multiple relevant topics were assigned to all applicable categories for further analysis. The categories were as follows: absolute monocyte count (AMC), LMR, MLR, irAEs, monocyte markers, and monocytic myeloid-derived suppressor cells (m-MDSCs). Although tumor-associated macrophages (TAMs) play a crucial role in cancer progression and are targeted by many biologics to potentiate ICI efficacy, we excluded them from our search because they require biopsies [21]. At this step, irrelevant articles were also excluded. There was no age/gender/type of cancer/number of metastasis restriction for the inclusion process.

### Data analysis

For efficacy measurement, we used hazard ratios (HR) of progression-free survival (PFS) and overall survival (OS), with 95% confidence intervals (CI). We used the odds ratio (OR) of the occurrence of irAEs for safety assessment. When HR and OR were selected as outcome measurements, natural logarithm transformation was applied for meta-analysis. In studies where HR and CI were not reported, the data was calculated from the available data or extracted from figures in the articles. Further, the results were exponentiated for the presentation (Appendix 1.4) [22]. If a publication included univariate (UV) and multivariate (MV) analyses, data were extracted and analyzed separately. We performed a Bayesian meta-analysis, as the included studies were heterogeneous, and some had low observations and wide CIs. This method allowed a better estimation of the actual population effect. Bayesian meta-analysis was applied even when data appeared homogeneous according to the test of residual heterogeneity. Posterior estimates of Bayesian meta-analysis per factor and the funnel plots were used to determine the publication bias. Meta-analysis was performed only when at least three studies examined a particular marker. For Bayesian meta-analysis, we used model averaging, Bayes Factor 10, H0, and H1 prior model probability set to 0.25, estimation sample settings set to 2000, and number of chains 4. Bayes factor computation method was integration. The meta-analysis includes fixed effect and random effect data.

Forest plots represent the estimated means and CI (Appendix 1.6). Otherwise, a narrative review was conducted (Appendix 1.7–1.8). The full statistical description is provided in Supplementary 1.4 and Table 1 [23]. The risk of bias assessment was done using the Cochrane-ROBINS-I tool (Appendix 1.9, 2) [24]. Only five articles were excluded based on the reporting bias (not included in the final analysis), as the data was controversial within the publication and, therefore, impossible to retrieve. Certainty assessment was conducted using the GRADE tool (Appendix 1.10, 2.6) [25]. We performed sensitivity and heterogeneity analyses by removing one study from each Bayesian meta-analysis and calculating Tau and random effects mean, median, and CI (Appendix 1.5, 1.6).

**Table 1:** Differentially expressed soluble monocyte serum markers in relation to ICI treatment outcome (combined data for melanoma, NSCLC, metastatic gastrointestinal tumors)

	Favorable soluble markers		Unfavorable soluble markers	
	High	Low	High	Low
Cytokine/chemokine	CCL18 [17]		CCL18 [110]	
	MCP-1 [115]		MCP-2 [114]	
	MCSF [17]		sCD163 [110]	
		Flt‐3L [17]		MCP-1 [111]
		CXCL1 [112]		
		CCL23 [17]		
		MCP-1 [112, 113]		

Blue—favorable outcomes include complete pathological response, durable clinical benefit, and longer PFS. Red—unfavorable outcomes, such as hyper-progressive disease, shorter PFS, and no response. *CCL18* chemokine (C–C motif) ligand 18, *MCP-1* monocyte chemoattractant protein-1, *MCSF* macrophage colony-stimulating factor, *Flt-3L* FMS-like tyrosine kinase three ligands. *CXCL1* chemokine ligand 1, *CCL23* chemokine (C–C motif) ligand 23, *MCP-2* monocyte chemotactic protein-2, *sCD163* soluble CD163.

### Role of the funding source

There was no funding source for this study.

## Results

Out of 5787 non-duplicated records identified in our search, 155 eligible studies reported monocyte-related markers as predictors of response to ICI, and 32 described a relationship to irAEs. Based on the reporting bias, we excluded only five studies as they contained conflicting information (the direction of effect varied within the text) and data that could not be reliably extracted (Fig. 1). All relevant studies were subdivided into categories depending on the publication’s focus: AMC, LMR, MLR, monocyte markers, m-MDSCs, and irAEs. Appendix 3 presents the extracted data from the systematic review regarding the cancer type and therapy administered. Figure 1 presents the study selection diagram.

### Absolute monocyte count

**Fig. 2:**
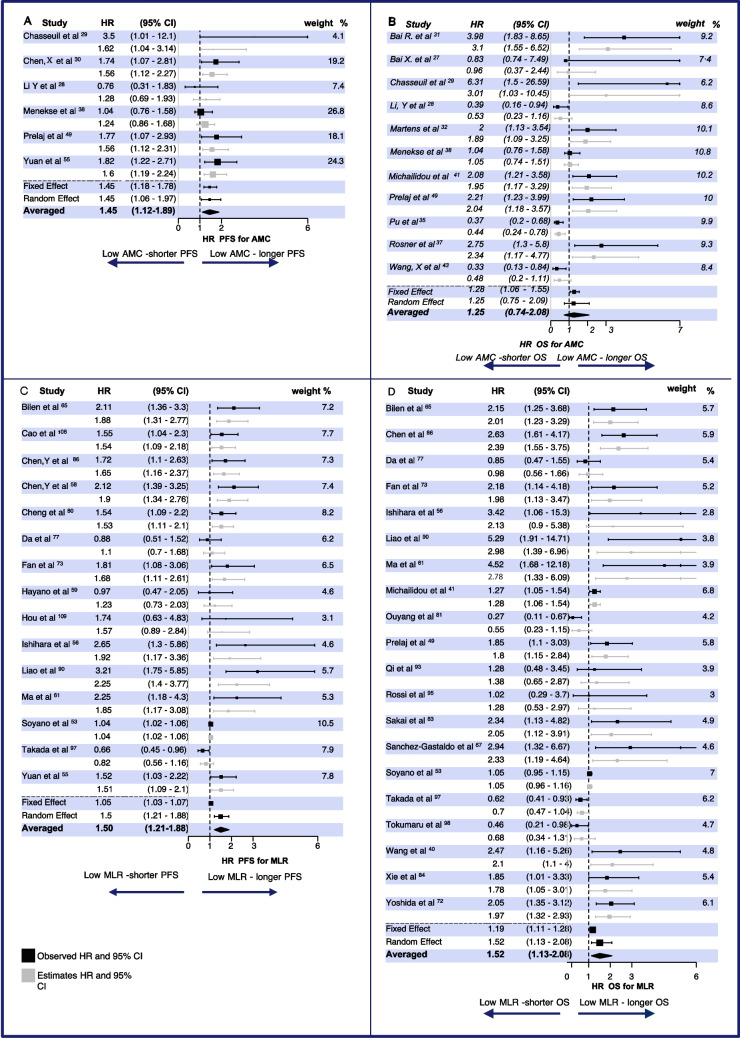
Meta-analysis for absolute monocyte count (**A**, **B**) and monocyte lymphocyte ratio (**C**, **D**) as predictors of overall survival (OS) and progression-free survival (PFS) in patients receiving immune checkpoint inhibitors (ICIs). **A** HR PFS for AMC. **B** HR OS for AMC. **C** HR PFS for MLR. **D** HR OS for MLR. Fixed effect data is presented in black symbols/intervals, and Bayesian estimated effect in grey symbols/intervals

Thirty articles investigated the predictive value of baseline absolute monocyte count (AMC) for the efficacy of ICIs [26–57]. Thirteen studies examined AMC’s role in predicting PFS [26–30, 38, 49, 52–57]. Of these six, reported significantly longer PFS in patients with low AMC in univariate analysis (UA) (Appendix 4, 6) [29, 30, 49, 53, 55, 57], while six others—no association [26–28, 52, 54, 56]. Multivariate analysis (MV) was not conducted in all studies but revealed a minor effect (Fig. 2A) [28, 29, 38, 49, 50, 55]. No significant differences were observed across cancer types or therapeutic targets (PD-1, PD-L1, CTLA-4, or combination therapy) (Appendix 6.1A, B).

Eight studies found no association between AMC and overall survival (OS) [26–28, 31, 35, 43, 54, 56], whereas eight others reported that high baseline AMC was associated with shorter OS [29, 32, 37, 41, 42, 47, 49, 53, 57] (Fig. 2B, Appendix 4). In contrast, four studies reported the opposite effect, associating low baseline AMC with poor prognosis [28, 35, 38, 43]. Bayesian meta-analysis of MV data showed no significant association between baseline AMC and OS with HR 1.25, 95% CI (0.7–2.08) (Fig. 2B). In most studies, MV HR was closer to 1 than UV analysis, showing that other variables could cause the UV effect. The median and interquartile range (IQR) for high versus low AMC cut-off values were 640 (IQR: 478–650) (Appendix 7). Two studies described the direction of the effect, but their numerical data did not support their conclusions [39, 46]. Most studies had moderate (35%) and serious (38%) risk of bias. PFS studies were more homogeneous than OS, and MV analysis was more sensitive for both outcomes (Appendix 9). UV analysis was mainly skewed by one publication [38].

### MLR and LMR as predictors of survival

Out of the 109 papers included in this review, 28 reported on the monocyte-to-lymphocyte ratio (MLR) [39, 41, 42, 45, 53, 56, 58–80], and 37 examined the lymphocyte-to-monocyte ratio (LMR) as a potential biomarker for predicting PFS and /or OS in patients treated with ICIs [26, 30, 36, 40, 47, 49, 55, 57, 73, 81–109]. For meta-analysis, LMR was converted into MLR, and further results are presented for the pooled data. An improved PFS (HR 1.5, 95% CI 1.21–1.88), as well as OS (HR 1.52, 95% CI 1.13–2.08), were observed in patients with lower MLR, indicating that patients with low monocytes and (or) high lymphocytes were more likely to benefit from ICI (Fig. 2C, D, Appendix 5, 6). There were no significant differences between cancer types or therapeutic targets (PD1, PD-L1, CTLA-4, and combination) (Appendix 6.1C, D). However, for combined therapy, the evidence of low MLR favoring prognosis was more substantial. Regarding the cancer type, the OR for MLR for both PFS And OS was less in gastric and esophageal cancers than in other malignancies. The mean cut-off values were 2.56 for high vs. low LMR and 0.39 for MLR (Appendix 7).

Sekine and colleagues stated that the reduction of LMR is a favorable marker of objective response rate [96]. Another study reported that patients with LMR < 4.15 have an increased risk of progression and death compared to those with high LMR [100]. Zhu and colleagues reported that patients with high MLR had a shorter time to progression [69]. Four studies associated high baseline MLR with a lack of response to ICI therapy [39, 70, 89, 97], while five others showed no association (Appendix 5, 6) [66, 68, 71, 85, 94]. Zhang and colleagues suggested that MLR could be used to predict the pathological tumor regression grade [109]. Collectively, these data imply that low MLR is a favorable prognostic marker of therapy outcome. Most of the studies had a moderate (52%) risk of bias, with PFS being more sensitive and homogeneous than OS data (Tau < 0.4 vs Tau < 0.6) when removing publications one by one.

### *Monocyte markers*

#### Soluble monocyte-related markers

Seven studies focused on soluble monocyte-related markers, including MCSF, Flt-3L, CXCL1, CCL23, MCP-1, MCP-2, soluble CD163, and CCL18 (Table 1) [17, 110–115]. The data were categorized based on favorable outcomes—such as complete pathological response, durable clinical benefit, and longer PFS—and unfavorable outcomes, including hyper-progressive disease, shorter PFS, and lack of response. Out of four studies, two NSCLC studies of PD-1 inhibitors reported low MCP-1 as a favorable predictive marker [112, 113]. In contrast, two other studies—one on melanoma (anti-PD-1 ± anti-CTLA-4) and one on gastrointestinal tract tumors (anti-PD-1)—reported MCP-1 as an unfavorable prognostic marker [111, 115].

## Monocyte (sub)populations and surface proteins

Thirty-two studies on ICI treatment examined 24 different monocyte populations [15–17, 32, 39, 76, 116–141], revealing contradicting results for some defined subpopulations. Specifically, Krieg and colleagues reported that an increased frequency of classical HLA-DR^+^ monocytes was associated with a better prognosis [15]. In contrast, Araujo and colleagues reported HLA-DR^+^ classical and non-classical monocytes as negative prognostic markers [138]. In all but one study [15], a high frequency of PD-1⁺ or PD-L1⁺ circulating monocytes was a negative prognostic marker [76, 116, 118, 126, 129, 133, 136]. Ando and colleagues reported a lack of association between PD-L1 expression and different monocyte subtypes but noted that OS was shorter in patients with increased frequency of PD-L1^+^ CD14^+^ monocytes. These authors also argued that this finding could result from a selection bias, as OS distribution varied among different diseases. Therefore, the frequency could be disease-specific rather than survival-specific [136].

Better outcomes were associated with a low frequency of other checkpoint-positive monocytes such as LAG3^+^, PD‐L2^+^, B7‐H3^+^, and CTLA‐4^+^ [16, 142]. The total percentage of CD14^+^ cells was a positive predictor for response, supporting the positive predictive capacity of MLR [15, 32, 135]. Table 2 summarizes all extracted monocyte-related variables. Sixty-one percent of the studies had a moderate risk of bias, 15% were critical, and 17%–serious.

**Table 2:** Monocyte subpopulation markers as predictors of ICI efficacy

	High (frequencies or expression levels)						Low (frequencies)	
	Favorable predictor			Study	Unfavorable predictor	Study		Study
Classical CD14 + CD16- monocytes **(frequencies)**	CD14^+^CD16b^−^HLA-DR^hi^			^15^	CD14^+^CD16^−^	^39,117,124,127^	CD14^+^CD16b^−^CTLA-4^+^ (Favorable marker)	^17^
	CD14^+^CD16^−^CD33^hi^ CD14^+^CD16^−^CD86^+^			^16^	CD14^+^CD16^−^HLA-DR^+^	^138^		
	cMonocyteD7/D0 divided by cMonocyte-PD-L1^+^D7/D0 > 1			^76^	CD14^+^CD16^−^PD-L1^hi^ (day7)	^76^		
	CD14^+^CD16^−^ CD33^+^HLA-DR^+^CD11b^+^CD11c^+^CD14^high^CD16^low^			^131^	CD14^+^CD16^−^PD-L1^+^	^124^		
Non-classical CD14dimCD16 + monocytes **(frequencies)**	CD14^dim^CD16^+^			^121,124,126,132,137^	CD14^dim^CD16^+^	^117^	CD14^dim^CD16^−^PD-L1^+^ (Favorable marker)	^126^
	CD14^dim^CD16^+^CD9^+^			^16^	CD14^dim^CD16^+^HLA-DR^+^	^139^	CD14^dim^CD16^+^SLAN^+^ (Unfavorable marker)	^125^
	CD14^dim^CD16^+^SLAN^+^			^123^	CD14^dim^CD16^+^PD-L1^+^	^124^	CD14^dim^CD16^+^ (Unfavorable marker)	^128^
	CD14^dim^CD16^+^HLA-DR^+ +^			^121^				
	CD33^+^ HLA-DR^+^CD11c^+^CD16^high^CD14^low^			^131^				
Intermediate CD14 + CD16 + monocytes	CD69 **(expression)**			^17^	CD14^+^CD16^+^ **(frequencies)**	^140^	CD14^+^CD16^+^PD-L1^+^ **(frequencies)** (Favorable marker)	^126^
Monocyte populations without subclassification **(frequencies)**	IPI-NIVO treatment CD14^+^CD11C^+^CD33^+^CD15^–^CD19^–^CD66B^–^PDL1^–^PDL2^+^CD163^+^GAL9^–^CD80^–^CD86^–^41BBL^+^CD40^+^OX40L^+^			^134^	NIVO-IPI treatment CD14^+^CD11C^+^CD33^+^CD15^–^CD19^–^CD66B^–^PDL1^–^PDL2^+^CD163^+^GAL9^–^CD80^–^CD86^–^41BBL^+^CD40^+^OX40L^+^	^134^	LAG3^+^ PD‐L2^+^ B7‐H3^+^ CTLA‐4^+^ (Favorable marker)	^142^
	CD14^+^			^15,32^	PD-L1^+^	^133,136^		
	CD14^++^			^135^	FKBP51s^+^ PD-L1^+^	^133^		
	CD16^hi^			^132^	MSR1^+^	^133^		
	CD14^+^interferon response genes carrying phenotype			^119^	PD-1^+^	^116^	PD-L1^+^ (Unfavorable marker)	^120^
	CD14^+^Slan^+^			^130^	HLA-DR^+^ CD16^+^	^116^		
	CD14^+^ HLA-DR^+ +^			^122^	pAkt (intracellular)	^139^		
Monocyte marker **expression levels**	SLAN IgE CD62L	^16^	CD33 CD11B CD303 CD62L CD1c CD64 CD14 CD34	^15^	PD-L1	^117,139^		
	CD86	^15,16^						
	HLA-DR CD141 ICAM-1 CD11C PD-L1 CD38 CD16	^15^						

Blue—favorable outcomes, such as complete pathological response, durable clinical benefit, and longer PFS. Red—unfavorable outcomes, such as hyper-progressive disease, shorter PFS, and no response. Monocyte populations are presented as classical, intermediate, non-classical, and unclassified, along with markers that were shown to be differentially expressed on monocytes in terms of intensity.

Beyond flow cytometry phenotyping, four reports covered RNA-sequencing data, revealing 86 differentially expressed monocyte genes between patients who benefited from ICI therapy and those who did not (Appendix 8) [15, 76, 105, 133]. Several gene ontology patterns were associated with unfavorable outcomes, including neutrophil and monocyte migration, neutrophil aggregation, RAGE receptor binding, toll-like receptor 4 binding, chemotaxis, and response to hydrogen peroxidase.

### Monocytic myeloid-derived suppressor cells (m-MDSCs)

Among 21 studies focusing on m-MDSCs, 16 reported that low m-MDSCs frequencies were common in patients with improved PFS and OS (Table 3) [28, 44, 57, 126, 130, 137, 138, 143–156]. Despite variations in gating strategies across studies, 13 reported low baseline m-MDSCs as positive predictive markers of response to ICI (Table 3, Appendix 10). Three studies described the reduction of m-MDSCs after ICI as a positive predictive marker of improved PFS [144, 147, 153].

**Table 3:** m-MDSC-related markers as predictors of ICI efficacy.

m-MDSC markers					
Favorable predictor			Unfavorable predictor		
Parameter	Study	*N* patients/diagnosis	Parameter	Study	*N* patients/diagnosis
Baseline low % m-MDSCs	^32, 57, 123, 126, 130, 138, 143, 148, 154–156^	^392/melanoma,^ ^57/NSCLC,^ ^137/GITC,^ ^33/mixed^	High intracellular NO	^44^	^59/melanoma^
Week 3 low % m-MDSCs	^44, 146, 155^	^112/melanoma,^ ^61/NSCLC^	Low eotaxin-1		
Week 9 low % m-MDSCs	^146^	^43/melanoma^	High serum S100A8/A9		
m-MDSC reduction	^123, 144, 147^	^141/urothelial carcinoma,^ ^35/melanoma,^ ^23/NSCLC^	High serum HMGB1		
Low % PD-1^+^/PD-L1^+^ m-MDSCs	^145^	^41/melanoma^	High % HLA-DR^low^ m-MDSCs	^130^	^40/NSCLC^
cFLIP expression	^137^	^34/NSCLC^	High galectin-9^+^	^155^	^61/NSCLC^
Baseline high % m-MDSCs	^151^	^46/melanoma^	High PD-L1 m-MDSCs	^126^	^36/melanoma^
5 miRs present	^150^	^49/melanoma^			

Changes/reduction of the delta m-MDSCs between different time points: low % indicates decreased %(percentage) of m-MDSCs at different time points. Blue: favorable outcomes, such as improved PFS and OS, relapse-free survival, clinical benefit, and longer PFS. Red: unfavorable outcomes, such as shorter PFS and no response. The mean number of patients per publication was 54, with an SD of 28.

*cFLIP*: cellular FLICE (FADD-like interleukin-1β-converting enzyme)-inhibitory protein; *HMGB1*: high mobility group box 1; *S100A8/A9*: calcium- and zinc-binding proteins; *NSCLC* non-small cell lung cancer; *GITC* gastrointestinal tract cancer.

Notably, one study observed a transient increase in m-MDSC frequency 3 weeks after therapy initiation in patients who achieved a complete response, followed by a decline by week nine [149]. Cut-off values vary between studies, with a mean of 6.45(SD = 5.02), making it difficult to establish a definitive threshold. Regardless of the absolute cell numbers, low frequencies of m-MDSCs were consistently observed in patients benefiting from ICI. Only Tomela and colleagues reported the opposite effect [151].

M-MDSC phenotypes may also serve as prognostic markers in cancer patients receiving ICIs (Table 3). Galectin-9, PD-1, PD-L1, and increased intracellular nitric oxide (NO) were unfavorable predictors [44, 126, 155]. Adamo and colleagues reported FLIP positivity only in non-progressive patients [137]. Lastly, a significant association was shown between m-MDSC-derived microRNA and ICI-therapy outcome: patients who had no or only one of the miRNAs from a particular set (let-7e, miR-125a, miR-99b, miR-146b, and miR-125b) before immunotherapy had better PFS and OS [150]. Regarding the secretome, S100A8/A9 and high-mobility group box 1 (HMGB1) were linked to poor prognosis in ICI-treated patients [44]. Among the included studies, half had a moderate risk of bias, 30% were classified as severe, and only 5% as critical.

## Immune-related adverse events and monocyte markers

**Fig. 3:**
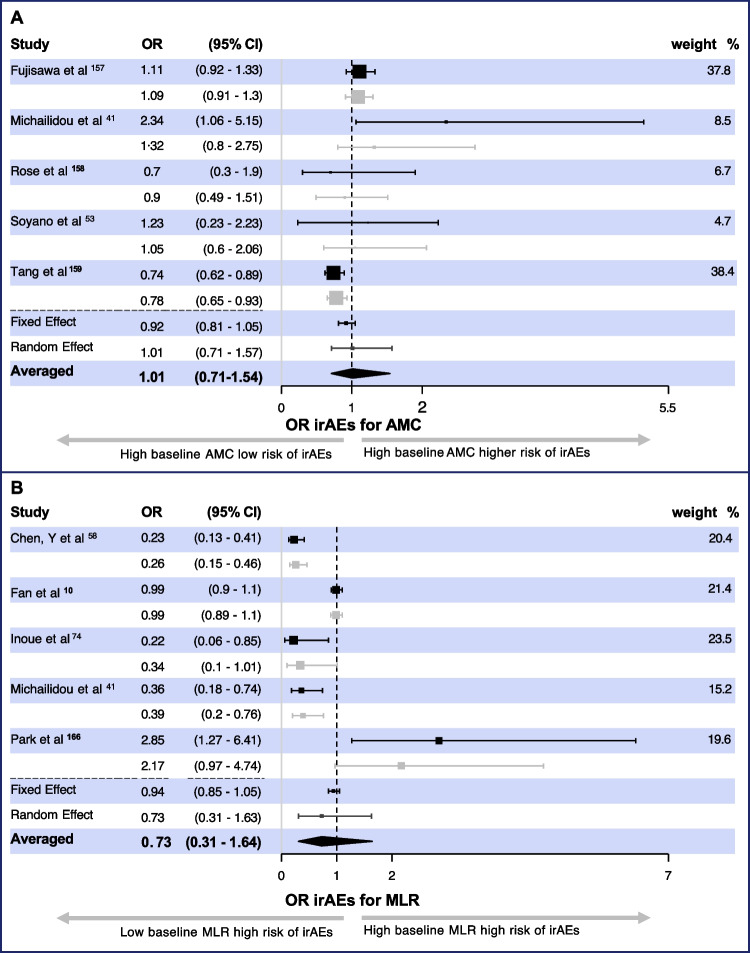
**A** Odds ratio of absolute monocyte count as a predictor of immune-related adverse events. **B** Odds ratio of monocyte/lymphocyte ratio (MLR) a predictor of immune-related adverse events. Black lines/symbols represent the observed effect reported in the studies, and grey lines/symbols represent the Bayesian estimated OR and 95% Cis. The weights are from the random effects model

Predicting irAEs and their severity remains a significant challenge in managing ICI-treated patients. Thus, new predictive biomarkers may help to adjust administration schedules and make dose adjustments in patients predicted to have high-grade, potentially life-threatening irAEs. Of the 32 articles studying the relation between baseline monocyte counts and related markers and the occurrence of any irAEs, ten covered AMC as a potential predictor. However, only five provided OR and CI data suitable for meta-analysis (Fig. 3A) [41, 53, 157–159]. Four studies mentioned increased numbers of monocytes in irAEs; however, OR were not presented [160–163].

Wölffer and colleagues reported that high total monocyte count was associated with colitis, hepatitis, or pancreatitis, and increased monocyte frequencies were only associated with colitis or pancreatitis [161]. Three other melanoma studies found no association between AMC and the development of irAEs [32, 164, 165]. It should be mentioned that these four publications differed in treatment protocols in CI and combinations. However, based on heterogeneity tests, the reports were homogeneous (Appendix 9). A preliminary conclusion is that absolute monocyte count is not significantly associated with irAE development (OR = 1.01, 95% CI: 0.71–1.54).

Five studies discussed the role of MLR [10, 41, 58, 74, 166]. Two studies examined the role of LMR [167, 168] in predicting irAEs. Our analysis suggests a tendency toward reduced risk of irAEs in patients with high baseline MLR OR 0.73 95% CI (0.3–2.3). However, the Bayesian odds ratio was not significant (Fig. 3B). The Tau value was affected by one publication [168]. And as the transformation of OR for LMR into MLR introduces wide CI, we excluded this study from the final meta-analysis. Zamora and colleagues discussed the predictive potential of monocyte-platelet (PLT) complexes in combination with lymphocyte-PLT complexes; higher frequencies of these complexes indicated an increased risk of dermatological irAEs [169].

**Fig. 4:**
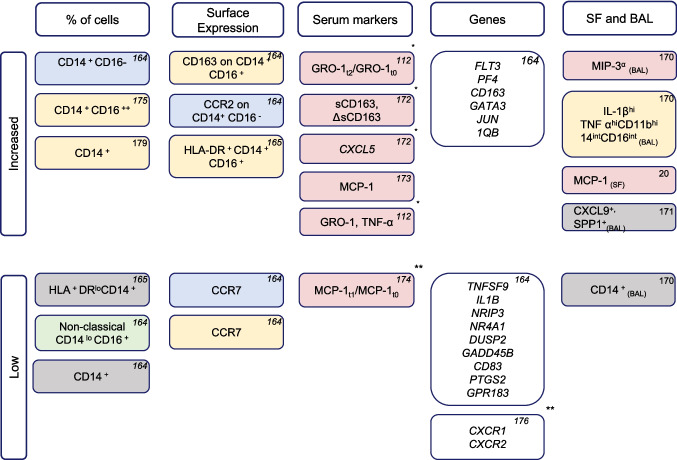
Monocyte markers associated with any irAEs. Blue: classical monocytes, green: non-classical monocytes, yellow: intermediate monocytes, grey: monocytes without subclustering, pink: soluble factors, and white: genes. Every block depicts one study and one parameter. The parameter is within “high” if the authors reported elevated levels of the parameter either compared to healthy controls, patients with non-ICI induced autoimmune conditions, or patients who did not develop irAEs. The parameter is within “low” if the authors reported lower levels of the parameter compared to healthy controls, patients with non-ICI-induced autoimmune conditions, or patients who did not develop irAEs. **studies with *n* > 100 participants; *studies with *n* 40–100 participants; no *studies with *n* < 30 participants

Several monocyte surface markers were significantly upregulated post-treatment in monocytes and were associated with irAE development (Fig. 4). These included CD14, CD16, CCR2, CCR7, and CD163 [164, 165, 170, 171]. Furthermore, chemokines such as CXCL5, sCD163, CXCL1, MCP-1, and GRO-1 were also statistically significantly higher [112, 172–174]. Besides the specific cell markers and chemokines, higher frequencies of intermediate and classical monocytes were also observed in patients who developed irAEs compared to those who did not [164, 165, 175]. Several gene expression levels were also higher in monocytes associated with the development of irAEs. These genes included *FLT3*, *PF4*, *CD163*, *GATA3*, and *JUN* [164]. Ye and colleagues reported low *CXCR1* and *CXCR2* in irAE patients [176]. One study did not draw any conclusions about the predictive power of these monocyte-relate markers [177]. Lepper and colleagues found no differences in monocytic PD-L1 and CD73 levels [178].

Conversely, some monocyte-related parameters were reduced in patients who developed irAEs. These parameters included a lower number of CD14^+^HLA-DR^low/neg^ monocytes (mMDSCs), decreased MCP-onefold change over time, and reduced frequency of non-classical monocytes. The reduction of these markers suggests a potentially impaired monocyte-related suppressive function in irAEs [20, 174]. Similarly, several genes were downregulated in monocytes associated with the development of irAEs (Fig. 4). Sixty-nine percent of the studies had a moderate risk of bias, 13% – serious, and 9% – critical. Only 14 publications reported both irAEs and survival, with MLR being the most frequently reported predictor [41, 74]. Four publications reported no correlation between irAEs and survival [10, 32, 96, 164], while ten others showed improved OS in patients with irAEs (Appendix 11) [41, 53, 58, 74, 112, 158, 174, 176, 178, 179].

## Discussion

This systematic review addresses a critical question in cancer immunotherapy: the identification of reliable predictive markers for ICI response and efficacy, as well as irAE development. Whereas most studies focus on lymphocyte-related parameters, we concentrate on monocyte-related parameters as possible predictors of treatment outcomes. Peranzoni and colleagues previously narratively reported some of our findings, such as the role of m-MDSCs and MLR, but did not conduct a systematic review and meta-analysis [4]. To our knowledge, this is the first study to comprehensively summarize monocyte-related blood biomarkers—such as AMC, MLR, m-MDSCs, and monocyte phenotypes—in the context of ICI treatment response and irAEs. Further prospective research is necessary for a complete understanding of the role of monocytes in irAEs, both in terms of toxicity management and diagnostics.

The results of this meta-analysis suggest that monocyte-related markers are potentially helpful predictive markers for ICI response, survival outcome, and safety. High MLR is associated with shorter PFS and OS, and high AMC and high frequencies of M-MDSCs tend to be unfavorable predictors. HLA-DR, CD86, and PD-L1 expression on monocytes might have a predictive value, but this would require more studies/data to substantiate. Additionally, monocyte profile can aid in selecting combination therapy vs monotherapy or in the discussion of refraining from more systemic treatment.

Although we were able to conduct a meta-analysis for AMC and MLR, the number of studies that performed multivariate analysis was limited. Considering the discrepancies between HR in multivariate and univariate analysis (UV), UV analysis introduces bias and should be avoided. We noted that MLR can be used as a prognostic marker for PFS and OS in ICI therapy. Still, many studies use neutrophil-to-lymphocyte ratio (NLR) or neutrophil-to-eosinophil ratio (NER), which may be a better prognostic marker for efficacy [180, 181]. Studies where MLR was part of a more complex predictor were excluded from our analysis [182]. Monocyte-related predictors can be combined with other clinical features and host-related and tumor-related biomarkers [13]. Therefore, while our findings suggest a potential role for MLR and monocyte phenotypes, we cannot rule out that combining blood-derived variables could enhance their predictive power. However, this question requires separate analyses to assess specificity and sensitivity.

Activated monocytes have dual functions: on the one hand, they can present tumor antigens, recruit natural killer cells, and maintain a proinflammatory environment, thereby fighting the cancer cells; on the other hand, by expressing checkpoint molecules and releasing immune suppressive factors, they can play a crucial role in cancer immune evasion, potentiating metastasis and tumor growth [4]. Given that monocytes orchestrate immune homeostasis and comprise different subpopulations (pro-tumorigenic and anti-tumorigenic), we suggest that more in-depth monocyte phenotyping has the potential use in predicting the response to ICI.

Despite the known physiological differences between classical, non-classical, and intermediate monocytes, we did not identify distinct phenotypes specifically related to favorable or unfavorable outcomes, primarily due to the limited number of studies. However, a trend showed that high frequencies of classical monocytes were associated with a worse prognosis, whereas high frequencies of non-classical monocytes showed the opposite effect. Additionally, high surface expression of checkpoint molecules was linked to poorer prognosis. Notably, low frequencies of checkpoint-positive monocytes (PD-1, PD-L1, LAG3, PD‐L2, B7‐H3, and CTLA‐4) were associated with improved outcomes [142]. Several markers, such as HLA-DR or MCP-1, showed contradictory prognostic values, favorable in some studies and unfavorable in others.

Additionally, we could not identify any monocyte-specific transcriptomic patterns associated with response, though the limited number of studies and small patient cohorts may have contributed to this uncertainty. Most of the studies in which monocyte phenotyping was conducted did not report whether a multivariate analysis was performed. Therefore, it was impossible to systematically assess the exact prognostic potential of monocyte phenotyping in ICI therapy. Our findings suggest that monocyte-related markers may be useful predictors of ICI efficacy, particularly in combination with other predictors. For instance, the role of dendritic cells (another myeloid cell subset that may also be derived from monocytes), as well as natural killer (NK) cells, was recently discussed in response to ICI [183, 184]. Further research is needed and encouraged to extend our findings and to understand the underlying mechanisms of monocyte involvement in the efficacy and safety of ICI.

Since ICIs disturb immune tolerance and trigger irAEs, it is also important to identify predictors of the safety profile. Finding reliable predictors of severe irAEs is critical, as these irAEs frequently require treatment interruption or permanent discontinuation, hospital admission, and prolonged (high-dosed) immunosuppressive therapy, which may lead to poor outcomes [185–187].

The field of irAEs is emerging and needs new therapeutic targets for managing irAEs. The number of studies covering the predictive capacity of monocytes for irAEs was limited; therefore, we could not draw any definitive conclusions. Most interestingly, the frequency of intermediate monocytes appeared to be more related to the occurrence of irAEs. Additionally, CD163 and MCP-1 in the inflammatory lesions and the fold change upon ICI treatment in expression levels on monocytes in blood tended to be associated with ICI-induced autoimmune conditions [20, 164, 170, 172].

Regarding the transcriptional profile, the downregulation of *TNFSF9*, *IL1B*, *NRIP3*, *NR4A*, *DUSP2*, *GADD45B*, *CD83*, *PTGS2*, *GPR183*, *CXCR1*, and *CXCR2* suggested potential alterations in monocyte function and activation in the context of irAEs [164]. It may prove highly informative to determine whether these findings are specific to ICI-induced autoimmune conditions or are common in the corresponding autoimmune non-ICI-induced diseases. These findings may also point to novel therapeutic targets for the latter.

Despite the insights of our systematic review and meta-analysis, several limitations should be acknowledged. The variability in study designs, including differences in methodologies, patient populations, and treatment regimens, may introduce heterogeneity into our analysis. We did not subdivide studies into different ICI subgroups, as we focused on identifying universal markers. Secondly, the utility of predictors across all tumor types remains a subject for consideration, as the predictive power of MLR may vary depending on the specific cancer type. A significant challenge in using monocyte-related biomarkers in clinical practice is the lack of standardized cut-off values for AMC, MLR, and m-MDSCs. Different studies used centre-specific thresholds. Future prospective studies should establish uniform cut-off values to enhance reproducibility and clinical utility. Moreover, the number of studies reporting multivariate analysis was low, which might exaggerate the real population effect.

For the irAEs, it is also important to mention that the number of studies is limited. While this review highlights associations between monocyte-related markers and ICI outcomes, causal mechanisms remain unclear. The observed relationships do not establish whether monocyte subpopulations actively drive treatment response and autoinflammation or reflect broader immune dynamics. Future research should incorporate functional studies, such as single-cell RNA sequencing, mechanistic assays, and preclinical models, to determine how specific monocyte subsets contribute to ICI efficacy and toxicity.

In conclusion, in this systematic review and meta-analysis, we investigated the prognostic potential of monocyte-related markers in ICI therapy response, survival outcomes, and safety. Specifically, our results suggest that several monocyte markers, such as baseline MLR and m-MDSCs, are reliable predictors of PFS and OS. Monocyte phenotyping may be helpful, although further research is needed to uncover the underlying mechanisms related to disease outcome and irAE development.

## Supplementary Information

Below is the link to the electronic supplementary material.

Supplementary file1 (PDF 1698 kb)

## Data Availability

The supplementary appendix contains all extracted data, scripts, numbers, and quality assessments.
