# Supplementary material for: Monocyte-related markers as predictors of immune checkpoint inhibitor efficacy and immune-related adverse events: a systematic review and meta-analysis
Source: Cancer Metastasis Rev. 2025 Feb 21;44(1):35. doi: 10.1007/s10555-025-10246-6 (PMC11845441; doi:10.1007/s10555-025-10246-6)
Supplement: Supplementary file 1 — Supplementary file1 (PDF 1698 kb) [file 10555_2025_10246_MOESM1_ESM.pdf]

Monocyte-related markers as predictors of immune checkpoint inhibitors efficacy and immune-related adverse events: a systematic review and meta-analysis. Supplementary data.

Table of Contents

1. **Methods** 2
   1.1 Databases and keywords 2
   1.2 Inclusion criteria and abstract screening. 3
   1.3 Data extraction and full-text screening. 3
   1.4 Statistical analysis 3
   1.5 Sensitivity and heterogeneity analysis 4
   1.6 MetaBMA R_script with settings 4
   1.7 Gene analysis 4
   1.8 Methodology regarding specific monocyte-related markers. 5
   1.9 Risk of bias 5
   1.10 Certainty assessment for outcomes 5
2. **Risk of bias assessment** 7
   2.1 AMC 7
   2.2 Monocyte Markers 8
   2.3 MLR 9
   2.4 m-MDSCs 10
   2.5 irAEs 11
   2.6 GRADE certainty criteria 12
3. **Included articles and extracted data** 13
4. **HR PFS and OS for absolute monocyte count (AMC)** 20
5. **HR PFS, OS and response for monocyte lymphocyte ratio (MLR)** 21
6. **Meta-analysis of univariate HR OS and PFS for AMC and MLR** 24
   6.1 lnHR OS and PFS for AMC and MLR, stratified by diagnosis and therapeutic target. 26
7. **Cutoff values for MLR and AMC** 27
8. **Monocyte genes as predictors of ICI response.** 28
9. **Sensitivity and heterogeneity analyses for random effects models of Bayesian meta-analysis** 29
10. **Gating strategies for mMDSCs stratified by the reported effect and diagnosis** 32
11. **Studies discussing irAEs and survival outcomes.** 33

1. Methods

1.1 Databases and keywords.

The current systematic review followed the Preferred Reporting Items for Systematic Reviews and Meta-Analyses (PRISMA) statement (Additional file 3: Appendix C). To investigate the current knowledge on the role of monocytes in ICI treatment and irAEs, we searched three databases, PubMed, Embase, and Web of Science, for articles that were published from 2000 to December 2023 as the use of checkpoint inhibitors in humans wasn't reported before. Initial search was performed from January 2000 to October 2022, and another search was performed for the rest of 2022 and 2023. We aimed to review only human studies and placed no language restriction. However, from 4 non-English studies, we could not screen the full articles. This systematic review was registered with Prospero (registration number CRD42023396297 before conducting data extraction and analysis).

Firstly, to identify the role of the monocytes in the response to ICI, we performed a PubMed search (from 2000 until December 2023) with the next set of keywords: "X", "Y", and "Z". Where "X" represented known ICI, and molecules and targets and included key words: "Immune Checkpoint Inhibitors"[Mesh] OR (("Programmed Cell Death 1 Receptor"[Mesh] OR "CTLA-4 Antigen"[Mesh] OR "immune*"[tiab]) AND (inhibit*[tiab] OR "block*"[tiab] OR "antagonist*"[tiab])) OR "programmed cell death 1"[tiab] OR "programmed death 1"[tiab] OR "programmed death ligand 1"[tiab] OR "PD-1"[tiab] OR "PD1"[tiab] OR "PD-L1"[tiab] OR PDL1[tiab] OR "PDL-1"[tiab] OR "CD279"[tiab] OR "CD274"[tiab] OR "cytotoxic T-lymphocyte-associated protein 4"[tiab] OR "cytotoxic T-lymphocyte-associated antigen 4"[tiab] OR "CTLA-4"[tiab] OR "CTLA4"[tiab] OR "CD152"[tiab] OR atezolizumab[tiab] OR tecentriq[tiab] OR avelumab[tiab] OR bavencio[tiab] OR pembrolizumab[tiab] OR keytruda[tiab] OR durvalumab[tiab] OR imfinzi[tiab] OR ipilimumab[tiab] OR Yervoy[tiab] OR nivolumab[tiab] OR opdivo[tiab] OR cemiplimab[tiab] OR atezolizumab[tiab] OR avelumab[tiab] OR pembrolizumab[tiab] OR durvalumab[tiab] OR ipilimumab[tiab] OR nivolumab[tiab] OR cemiplimab[tiab]. All cancer types and metastasis terms were represented by "Y": "Neoplasms"[Mesh] OR "adenoma*"[tiab] OR "anticarcinogen*"[tiab] OR "blastoma*"[tiab] OR "cancer*"[tiab] OR "carcinogen*"[tiab] OR "carcinom*"[tiab] OR "carcinosarcoma*"[tiab] OR "chordoma*"[tiab] OR "germinoma*"[tiab] OR "gonadoblastoma*"[tiab] OR "hepatoblastoma*"[tiab] OR "hodgkin*"[tiab] OR "leukemi*"[tiab] OR "lymphangioma*"[tiab] OR "lymphangiomyoma*"[tiab] OR "lymphangiosarcoma*"[tiab] OR "lymphom*"[tiab] OR "malignan*"[tiab] OR "melanom*"[tiab] OR "meningioma*"[tiab] OR "mesenchymoma*"[tiab] OR "mesonephroma*"[tiab] OR "metasta*"[tiab] OR "neoplas*"[tiab] OR "neuroma*"[tiab] OR "nsclc"[tiab] OR "oncogen*"[tiab] OR "oncolog*"[tiab] OR "paraneoplastic*"[tiab] OR "plasmacytoma*"[tiab] OR "precancerous*"[tiab] OR "sarcoma*"[tiab] OR "teratocarcinoma*"[tiab] OR "teratoma*"[tiab] OR "tumor*"[tiab] OR "tumour*"[tiab]. Finally, "Z" represented monocytes "Monocytes"[Mesh] OR "monocyte*"[tiab]. Animal studies, reviews, and editorial letters were excluded using the following combination of keywords: NOT ("Animals"[Mesh] NOT "Humans"[Mesh]) AND NOT ("Letter"[Publication Type] OR "Editorial"[Publication Type] OR "Comment"[Publication Type]), AND NOT (("systematic review"[tiab] OR "systematic literature review*"[tiab] OR "Review"[Publication Type] OR "Meta-Analysis as Topic"[Mesh] OR "meta-analysis"[tiab] OR "Meta-Analysis"[Publication Type]).

Secondly, we performed an Embase search (from 2000 until December 2023) with the next set of keywords: "X" and "Y" and "Z". Where "X" and "Y" and "Z" represented the same aspects of the search. Thus "X" included: 'immune checkpoint inhibitor'/exp OR (('programmed death 1 receptor'/exp OR 'cytotoxic T lymphocyte antigen 4'/exp OR 'immune*':ti,ab,kw) AND (inhibit* OR 'block*' OR 'antagonist*'):ti,ab,kw) OR ('programmed cell death 1' OR 'programmed death 1' OR 'programmed death ligand 1' OR 'PD-1' OR 'PD1' OR 'PD-L1' OR PDL1 OR 'PDL-1' OR 'CD279' OR 'CD274' OR 'cytotoxic T-lymphocyte-associated protein 4' OR 'cytotoxic T-lymphocyte-associated antigen 4' OR 'CTLA-4' OR 'CTLA4' OR 'CD152' OR atezolizumab OR tecentriq OR avelumab OR bavencio OR pembrolizumab OR keytruda OR durvalumab OR imfinzi OR ipilimumab OR Yervoy OR nivolumab OR opdivo OR cemiplimab OR atezolizumab OR avelumab OR pembrolizumab OR durvalumab OR ipilimumab OR nivolumab OR cemiplimab):ti,ab,kw. "Y" represented: 'neoplasm'/exp OR ('adenoma*' OR 'anticarcinogen*' OR 'blastoma*' OR 'cancer*' OR 'carcinogen*' OR 'carcinom*' OR 'carcinosarcoma*' OR 'chordoma*' OR 'germinoma*' OR 'gonadoblastoma*' OR 'hepatoblastoma*' OR 'hodgkin*' OR 'leukemi*' OR 'lymphangioma*' OR 'lymphangiomyoma*' OR 'lymphangiosarcoma*' OR 'lymphom*' OR 'malignan*' OR 'melanom*' OR 'meningioma*' OR 'mesenchymoma*' OR 'mesonephroma*' OR 'metasta*' OR 'neoplas*' OR 'neuroma*' OR 'nsclc' OR 'oncogen*' OR 'oncolog*' OR 'paraneoplastic*' OR 'plasmacytoma*' OR 'precancerous*' OR 'sarcoma*' OR 'teratocarcinoma*' OR 'teratoma*' OR 'tumor*' OR 'tumour*'):ti,ab,kw. And "Z" was 'monocyte'/exp OR ('monocyt*'):ti,ab,kw. NOT ([animals]/lim NOT [humans]/lim) AND [2000-2022]/py. Animal studies, reviews, editorial letters were excluded using the following combination of keywords: NOT ([animals]/lim NOT [humans]/lim), NOT ('chapter'/it OR 'conference abstract'/it OR

'conference paper'/it OR 'conference review'/it OR 'editorial'/it OR 'erratum'/it OR 'letter'/it OR 'note'/it OR 'review'/it OR 'short survey'/it OR 'tombstone'/it).

Thirdly we screened WEB OF SCIENCE with the next set of keyword: "X" = TS=((("immune*") AND (inhibit* OR "block*" OR "antagonist*") OR "programmed cell death 1" OR "programmed death 1" OR "programmed death ligand 1" OR "PD-1" OR "PD1" OR "PD-L1" OR PDL1 OR "PDL-1" OR "CD279" OR "CD274" OR "cytotoxic T-lymphocyte-associated protein 4" OR "cytotoxic T-lymphocyte-associated antigen 4" OR "CTLA-4" OR "CTLA4" OR "CD152" OR atezolizumab OR tecentriq OR avelumab OR bavencio OR pembrolizumab OR keytruda OR durvalumab OR imfinzi OR ipilimumab OR Yervoy OR nivolumab OR opdivo OR cemiplimab OR atezolizumab OR avelumab OR pembrolizumab OR durvalumab OR ipilimumab OR nivolumab OR cemiplimab). "Y" = TS=("adenoma*" OR "anticarcinogen*" OR "blastoma*" OR "cancer*" OR "carcinogen*" OR "carcinom*" OR "carcinosarcoma*" OR "chordoma*" OR "germinoma*" OR "gonadoblastoma*" OR "hepatoblastoma*" OR "hodgkin*" OR "leukemi*" OR "lymphangioma*" OR "lymphangiomyoma*" OR "lymphangiosarcoma*" OR "lymphom*" OR "malignan*" OR "melanom*" OR "meningioma*" OR "mesenchymoma*" OR "mesonephroma*" OR "metasta*" OR "neoplas*" OR "neuroma*" OR "nsclc" OR "oncogen*" OR "oncolog*" OR "paraneoplastic*" OR "plasmacytoma*" OR "precancerous*" OR "sarcoma*" OR "teratocarcinoma*" OR "teratoma*" OR "tumor*" OR "tumour*"), and "Z" = TS=("monocyt*") respectively. For this database automatic exclusion of animal studies wasn't possible, therefore the next set of exclusion keywords was used: and Review Articles or Proceedings Papers or Meeting Abstracts or Editorial Materials or Book Chapters or Notes or Letters or Retracted Publications or Corrections or Reprints (Exclude – Document Types). And Review Articles or Proceedings Papers or Meeting Abstracts or Editorial Materials or Book Chapters or Notes or Letters or Retracted Publications or Corrections or Reprints (Exclude – Document Types) and Immunology or Oncology or Cell Biology or Biochemistry Molecular Biology or Research Experimental Medicine or Hematology or Pharmacology Pharmacy or Science Technology Other Topics or Chemistry or Pathology or Rheumatology or Surgery or Toxicology or Physiology or Biophysics (Research Areas)) and 1999 or 1998 or 1997 or 1996 or 1995 or 1994 or 1993 or 1992 or 1991 or 1990 or 1985 or 1976 (Exclude – Publication Years).

1.2 Inclusion criteria and abstract screening.

All titles/abstracts identified in the electronic databases were screened by two authors (AE, MT) independently of one another to decide whether the studies meet the eligibility criteria for this review. Rayyan.ai tool was used for abstract screening.[1] Discrepancies were resolved by discussion; if not, the third author (FK) was asked for additional screening. Articles were included if there was an indication about cancer and/or ICI and monocytes or any monocyte-related terminology. Animal studies were excluded manually, and for studies where it was unclear whether the study was human or animal, the article was included for further full-text analysis. Also, studies where ICI were not used were excluded from further analysis.

1.3 Data extraction and full-text screening.

All potentially relevant full texts were screened by three authors (AE, MT, and FK). First, AE screened all articles and extracted the data, and then MT and FK independently screened the same set of articles. Discrepancies were resolved by discussion. Indications, number of patients, presence of control groups, monocyte markers, monocyte-related cytokines, monocyte/lymphocyte ratio (MLR) and lymphocyte/monocyte ratios (LMR), and the reporting of irAEs were extracted. Additionally, the type of the study (prospective or retrospective), blinded or open-label, randomised or not, was assessed as well as the given therapy, reported information on drop-outs and the given therapy. All included articles were subdivided into categories according to the main focus of the study. If the article contained more than one subject of interest, it was included in all categories for further data analysis. The categories were as follows: absolute monocyte count (AMC), LMR, MLR, irAEs, monocyte markers, and monocytic Myeloid-derived suppressor cells (m-MDSCs). At this step, irrelevant articles were also excluded, as well as articles where more than one biological therapy was used. There were no age, gender, type of cancer, or number of metastasis restrictions for the inclusion process. However, we excluded studies with multiple biological treatments, except two checkpoint inhibitors were administered.

1.4 Statistical analysis

We used hazard ratios (HR), 95% confidence intervals, number of participants, and p-values for efficacy measurement. When univariate (UV) and multivariate (MV) analyses were conducted in the publication, the data from both tests were extracted and analysed separately. In cases where data was presented only in figures, the Engauge Digitizer tool was used to get the numeric values from the graphs.[2] Hazard ratio was calculated for the studies, where only Kaplan-Meier curves were reported. Also, we used the previously described method to calculate the variance in cases where the median and range were measured.[3] All data were standardised, and if no confidence interval was mentioned, we used calculations described by Hebert and colleagues.[4] HR was selected as an outcome measurement, and the data was heterogeneous and non-normally distributed, so we implemented ln transformation for meta-analysis.[4] Afterwards, the estimated medians and confidence intervals were exponentiated and plotted in forest plot data for visualisation. Bayesian meta-analysis was used, even when the data were homogeneous according to the test of residual heterogeneity, and posterior estimates of

Bayesian meta-analysis per factor and the funnel plots were used to determine the publication bias. Meta-analysis was performed when the number of publications regarding particular marker reached three. For Bayesian meta-analysis, we used model averaging, Bayes Factor 10, H_0, and H_1 prior model probability set to 0.25, estimation sample settings set to 2000, and number of chains 4. Bayes factor computation method was integration. If other calculations were applied, it would be specified in the text. We used both fixed-effect and random-effect Bayesian models for presentation as they better characterise estimated publication bias. All statistical analyses were performed in R.[5] The MetaBMA package was utilised for data analysis with a model-averaging approach.[6] The full R-script is presented below.

1.5 Sensitivity and heterogeneity analysis

As Bayesian meta-analysis was conducted, we performed random effects Bayesian meta-analysis, removing one study at a time and calculated the mean, the median and 95%CI for Tau (the analog of I2 in Bayesian analysis) and the study estimates. When Tau is below 0.3, the heterogeneity is considered low, between 0.3-0.6 median, 0.6 to 1- moderate, and above 1- high. The full R-script is presented below.

1.6 MetaBMA R_script with settings

```
install.packages("metaBMA")
library("metaBMA")
library(readxl)
library(tidyr)
library(dplyr)
# Load data from CSV file (here the data is ln-transformed for HR
and OR and CIs, respectively)
data <- read_excel("path_to_data.xlsx")

#LnOR is the ln of the observed OR, STUDY column includes the
author data.
#check the data file
data
# Calculate standard errors from confidence interval limits

se <- (data$LnOR_Max - data$LnOR_Min) / (2 * qnorm(0.975))
# make sure the SE are all positive values and there are no typos in
CI values
se
# Bayesian Model-Averaged Meta-Analysis (H1: d>0)
set.seed(1) #try as well 123, this won't affect the result)

mb <- meta_bma(data$LnOR, se, data$STUDY, data,
        d = prior("cauchy", c(location = 0, scale = 0.707)),
        rscale_contin = 0.5,
        rscale_discrete = 0.707,
        prior = c(1, 1, 1, 1),
        tau = prior("invgamma", c(shape = 1, scale = 0.15)),
        ci = 0.95,
        logml_iter = 4000,
        chains = 4,
        logml = "integrate",
        summarize = "stan",
        thin = 1,  # Try different values, e.g., 2,
        iter = 2000,  # Try different values
        rel.tol = .Machine$double.eps^0.3)

mb
plot_forest(mb)
# (a) get fit from model above
mb_random <- mb$meta$random
# compute study weights
sigma <- mb_random$data$SE
tau <- mb_random$estimates["tau", "50%"]    # based on posterior
median
w <- 1 / (tau^2 + sigma^2)
weights <- w / sum(w)
#names(weights) <- mb_random$data$labels
Weights
# For sensitivity analysis
# Load data from CSV file
data_all <- read_excel("path_to_data.xlsx")
output <- data.frame(matrix(ncol = 9, nrow = 0))

# sampling a subtype for each loop
for (st in distinct(data_all, subtype, .keep_all=TRUE)$subtype){
  data_st <- data_all[data_all$subtype == st,]

 # dropping one sequential study in each loop
  for (i in 1:(dim(data_st)[1])){
    data_st_dropped <- data_st[-i,]

 # Calculate standard errors from confidence interval limits
    se <- (data_st_dropped$LnOR_Max -
data_st_dropped$LnOR_Min) / (2 * qnorm(0.975))

 #Bayesian Model-Averaged Meta-Analysis (H1: d>0)
    set.seed(1)
    mb_random <- meta_random(data_st_dropped$LnOR, se,
data_st_dropped$STUDY, data_st_dropped,
                d = prior("cauchy", c(location = 0, scale = 0.707)),
                #d = prior("norm", c(mean = 0, sd = 100)),
                summarize = "integrate",
                tau = prior("invgamma", c(shape = 1,    scale =
0.15)))
    # saving Tau and d
    data_st[i,'tau_mean'] <- mb_random$estimates["tau", "mean"]
    data_st[i,'tau_med'] <- mb_random$estimates["tau", "50%"]
    data_st[i,'tau_2.5%'] <- mb_random$estimates["tau", "2.5%"]
    data_st[i,'tau_97.5%'] <- mb_random$estimates["tau", "97.5%"]
    data_st[i,'rfs_mean'] <- mb_random$estimates["d", "mean"]
    data_st[i,'rfs_med'] <- mb_random$estimates["d", "50%"]
    data_st[i,'rfs_2.5%'] <- mb_random$estimates["d", "2.5%"]
    data_st[i,'rfs_97.5%'] <- mb_random$estimates["d", "97.5%"]
  }
  # appending subset's values to the output dataframe
  output = rbind(output, data_st)
}

write_xlsx(output, "tau_of_all_papers.xlsx")
```

1.7 Gene analysis

To identify genes that have any interaction, String database and PANTHER knowledge base were used to determine the main functions associated with given gene profiles, and Benjamini Hochberg correction was implemented for the functional analysis, as the initial number of articles was too low.[7]

1.8 Methodology regarding specific monocyte-related markers.

**Criteria**	**Method**
Absolute monocyte count (AMC)	Meta-analysis of the studies, where baseline AMC was reported in correlation with overall survival (OS), progression-free survival (PFS) and response.
Monocyte-to-lymphocyte ratio (MLR) and lymphocyte-to-monocyte ratio (LMR)	We transformed LMR to MLR and conducted a meta-analysis of the pooled data from studies where baseline MLR and LMR were reported in correlation with OS and PFS. MLR was calculated from LMR by dividing one by LMR.
Soluble monocyte-related markers	Since the number of studies was too low to do any statistical analysis, we distributed the markers according to their expression direction (reduced or increased) and whether they indicated a favourable or unfavourable prognosis. Where favourable was longer PFS and/or OS, and/or time to response, and/or response unfavourable is the opposite.
Monocyte populations and surface proteins	We selected the articles that looked at the frequencies of different monocyte populations. As the outcomes were too heterogeneous to conduct a meta-analysis, we focused on those parameters which revealed significant differences and then grouped these differences according to the effect direction (favourable or unfavourable prognosis). Where favourable was longer PFS and/or OS, and/or time to response, and/or response unfavourable is the opposite.<br>Classical monocytes were defined as $CD14^{+}CD16^{-}$<br>Non-classical monocytes were defined as $CD14^{dim}CD16^{+}$<br>Intermediate monocytes were defined as $CD14^{+}CD16^{+}$
Monocyte RNA-sequencing data	Genes were divided into genes with favourable and unfavourable prognostic properties. Where favourable was longer PFS and/or OS, and/or time to response, and/or response unfavourable is the opposite. String database and PANTHER knowledgebase were used to determine the main functions associated with given gene profiles. Benjamini Hochberg correction was implemented for the functional analysis, as the initial number of articles was too low.[7]
Monocytic Myeloid-derived suppressor cells (m-MDSCs)	We selected the articles that investigated m-MDSCs populations as well as total m-MDSCs. As the outcomes were too heterogeneous to conduct a meta-analysis, all m-MDSCs markers were extracted and grouped according to the effect direction (favourable, unfavourable or non-significant). Where favourable was longer PFS and/or OS, and/or time to response, and/or response unfavourable is the opposite.
Immune-related adverse events (irAEs) and AMC and MLR	Meta-analysis of the studies, where baseline AMC was reported in correlation with the development of irAEs<br>Meta-analysis of the studies, where baseline MLR was reported in correlation with the development of irAEs
Immune-related adverse events (irAEs) and monocyte markers	Selected the articles that looked at the frequencies of different monocyte populations and irAEs. As the outcomes were too heterogeneous to conduct a meta-analysis, markers were divided according to their direction (increased or low), type of markers such as frequency of the cells, expression levels, serum markers, genes and markers found in synovial fluid and bronchoalveolar lavage.<br>Classical monocytes were defined as $CD14^{+}CD16^{-}$<br>Non-classical monocytes were defined as $CD14^{dim}CD16^{+}$<br>Intermediate monocytes were defined as $CD14^{+}CD16^{+}$

1.9 Risk of bias

Risk of bias assessment was done using the Cochrane-ROBINS-I tool.[8] We conducted a risk of bias assessment for articles covering different categories separately, as the complexity and criteria sometimes differed. Also, the visualisation is provided separately for each of the subtopics. Some studies could have different scores in different categories, as the reporting bias and outcomes measurement could vary in the same publication.

1.10 Certainty assessment for outcomes

Certainty assessment was conducted using Grading of Recommendations Assessment, Development and Evaluation (GRADE) approach, using the following domains:
Risk of Bias, Inconsistency, Indirectness, Imprecision, Publication Bias
For each subcategory of this systematic review, the scoring was done separately, as the evidence varied between the subjects.[9]

References

1. Ouzzani M, Hammady H, Fedorowicz Z, Elmagarmid A. Rayyan—a web and mobile app for systematic reviews. Syst Rev. 2016 Dec 5;5(1):210.

2. Mark Mitchell BM and TW et al. Engauge Digitizer Software [Internet]. [cited 2023 Apr 11]. Available from: https://markummitchell.github.io/engauge-digitizer/

3. Hozo SP, Djulbegovic B, Hozo I. Estimating the mean and variance from the median, range, and the size of a sample. BMC Med Res Methodol. 2005 Apr 20;5(1):13.

4. Hebert AE, Kreaden US, Yankovsky A, Guo D, Li Y, Lee SH, et al. Methodology to standardise heterogeneous statistical data presentations for combining time-to-event oncologic outcomes. PLoS One. 2022 Feb 1;17(2):e0263661.

5. R: The R Project for Statistical Computing [Internet]. [cited 2024 Feb 13]. Available from: https://www.r-project.org/

6. metaBMA: Bayesian Model Averaging for Random- and Fixed-Effects Meta-Analysis [Internet]. [cited 2024 Feb 13]. Available from: https://cran.r-project.org/web/packages/metaBMA/vignettes/metaBMA.html

7. Szklarczyk D, Franceschini A, Wyder S, Forslund K, Heller D, Huerta-Cepas J, et al. STRING v10: protein-protein interaction networks, integrated over the tree of life. Nucleic Acids Res. 2014;43:447–52.

8. Sterne JA, Hernán MA, Reeves BC, Savović J, Berkman ND, Viswanathan M, et al. ROBINS-I: a tool for assessing risk of bias in non-randomised studies of interventions. BMJ. 2016 Oct 12;355.

9. Guyatt GH, Oxman AD, Vist GE, Kunz R, Falck-Ytter Y, Alonso-Coello P, et al. GRADE: an emerging consensus on rating quality of evidence and strength of recommendations. BMJ. 2008 Apr 26;336(7650):924–6.

2. Risk of bias assessment

**Bias due to confounding**	D1
**Bias in the selection of participants into the study**	D2
**Bias in the classification of Interventions**	D3
**Bias due to deviations from intended interventions**	D4
**Bias due to missing data**	D5
**Bias in the measurement of outcomes**	D6
**Bias in the selection of the reported result**	D7

2.1 AMC

**Study**	**D1**	**D2**	**D3**	**D4**	**D5**	**D6**	**D7**	**Overall bias**
Afzal et al (2019)	Serious	Low	Moderate	Low	Moderate	Low	Low	Serious
Bai R. et al (2021)	Moderate	Low	Moderate	Moderate	Low	Low	Moderate	Moderate
Bronte et al (2022)	Moderate	Low	Low	Low	Low	Moderate	Serious	Serious
Bai X. et al (2021)	Low	Moderate	Low	Low	Low	Low	Low	Low
Chasseuil et al (2018)	Low	Moderate	Low	Low	Serious	Moderate	Moderate	Moderate
Chen et al (2022)	Moderate	Moderate	Low	Low	Low	Low	Low	Moderate
Gebhardt et al (2015)	Moderate	Moderate	Low	Low	Moderate	Moderate	Serious	Serious
Goldschmidt et al (2023)	Low	Low	Moderate	Low	Low	Low	Low	Low
Juliá et al (2019)	Serious	Moderate	Low	Low	Serious	Critical	Serious	Critical
Khunger et al (2018)	Low	Moderate	Moderate	Moderate	Serious	Low	Low	Serious
Li, Y. et al (2022)	Low	Low	Moderate	Low	Low	Low	Low	Low
Martens et al (2016)	Moderate	Moderate	Low	Low	Low	Low	Low	Moderate
Menekse et al (2023)	Moderate	Moderate	Moderate	Moderatete	Low	Serious	Low	Serious
Okuhira et al (2018)	Moderate	Moderate	Moderate	Low	Moderate	Low	Low	Moderate
Parikh et al (2018)	Moderate	Moderate	Moderate	Low	Moderate	Low	Low	Moderate
Prelaj et al (2020)	Moderate	Low	Moderate	Low	Low	Low	Low	Low
Pu et al (2021)	Moderate	Moderate	Moderate	Low	Low	Serious	Low	Serious
Qi et al (2023)	Moderate	Moderate	Low	Low	Low	Low	Low	Moderate
Ribas et al (2016)	Serious	Moderate	Low	Low	Critical	Critical	Serious	Critical
Rosner et al (2018)	Moderate	Moderate	Moderate	Low	Low	Low	Low	Low
Shao et al (2021)	Moderate	Moderate	Low	Low	Low	Moderate	Moderate	Moderate
Soyano et al (2018)	Moderate	Serious	Moderate	Low	Moderate	Moderate	Low	Serious
Tanizaki et al (2018)	Moderate	Low	Low	Low	Moderate	Low	Low	Moderate
Wang, X et al (2023)	Low	Low	Low	Low	Low	Serious	Moderate	Serious
Wang, X. et al (2019)	Moderate	Low	Moderate	Low	Moderate	Serious	Low	Serious
Zheng et al (2023)	Serious	Serious	Low	Moderate	Low	Serious	Serious	Serious

2.2 Monocyte Markers

Study	D1	D2	D3	D4	D5	D6	D7	Overall bias
Adamo et al (2023)	Moderate	Low	Low	Low	Low	Low	Low	Low
Ando et al (2021)	Moderate	Critical	Low	Low	Low	Low	Moderate	Critical
B. de Lima et al (2021)	Moderate	Moderate	Low	Low	Low	Low	Critical	Critical
Comin-Anduix et al (2010)	Moderate	Moderate	Low	Low	Low	Moderate	Moderate	Moderate
Ende et al (2023)	Moderate	Low	Moderate	Low	Moderate	Low	Low	Moderate
Hofbauer et al (2022)	Moderate	Moderate	Moderate	Low	Low	Moderate	Low	Moderate
Hong et al (2022)	Moderate	Moderate	Low	Low	Low	Moderate	Critical	Critical
Hung et al (2021)	Moderate	Critical	Moderate	Low	Low	Moderate	Moderate	Critical
Jeon et al (2022)	Moderate	Moderate	Low	Low	Low	Low	Serious	Serious
Keenan et al (2022)	Moderate	Moderate	Low	Low	Low	Moderate	Low	Moderate
Krieg et al (2018)	Moderate	Low	Low	Low	Low	Low	Low	Moderate
Laza-Briviesca et al (2021)	Moderate	Low	Low	Low	Low	Low	Moderate	Moderate
Lee et al (2022)	Moderate	Moderate	Low	Low	Low	Low	Low	Moderate
Limagne et al.(2019)	Moderate	Low	Low	Low	Low	Moderate	Moderate	Moderate
Lo Russo et al (2023	Low	Low	Low	Low	Low	Moderate	Moderate	Moderate
Lu et al (2019)	Moderate	Moderate	Low	Moderate	Low	Critical	Critical	Critical
Ma et al (2023)	Moderate	Moderate	Low	Low	Low	Moderate	Low	Moderate
Martens et al (2016)	Moderate	Moderate	Low	Low	Low	Low	Low	Moderate
Möller et al (2022)	Moderate	Moderate	Low	Low	Low	Moderate	Low	Moderate
Nyakas et al (2019)	Moderate	Moderate	Low	Moderate	Low	Serious	Moderate	Serious
Ohkuma et al (2023)	Moderate	Low	Low	Low	Low	Serious	Low	Serious
Olingy et al (2022)	Moderate	Moderate	Low	Low	Low	Low	Low	Moderate
Oyanagi et al (2021)	Moderate	Low	Low	Low	Low	Moderate	Moderate	Moderate
Ozawa et al (2021)	Moderate	Moderate	Low	Moderate	Low	Low	Moderate	Moderate
Pedersen et al (2020)	Moderate	Low	Low	Low	Low	Low	Low	Moderate
Pettinella et al (2023)	Moderate	Moderate	Moderate	Low	Low	Moderate	Moderate	Moderate
Pico de Coaña et al (2020)	Moderate	Moderate	Low	Low	Low	Moderate	Low	Moderate
Pirozyan et al (2020)	Moderate	Moderate	Low	Low	Low	Low	Low	Moderate
Pour et al (2021)	Moderate	Moderate	Low	Low	Low	Moderate	Moderate	Moderate
Rapposelli et al (2021)	Serious	Serious	Low	Low	Serious	Serious	Serious	Serious
Riemann et al (2023)	Moderate	Moderate	Moderate	Low	Low	Moderate	Low	Moderate
Riemann et al (2020)	Moderate	Moderate	Low	Low	Low	Moderate	Low	Moderate
Rijnders et al (2023)	Low	Low	Low	Low	Low	Low	Low	Low
Rochigneux et al (2022)	Low	Moderate	Low	Low	Low	Moderate	Low	Moderate
Romano et al (2015)	Moderate	Moderate	Low	Low	Low	Serious	Moderate	Serious
Rossi et al (2022)	Moderate	Low	Low	Moderate	Low	Low	Low	Moderate
Shao et al (2021)	Moderate	Moderate	Low	Low	Low	Moderate	Moderate	Moderate
Troiani et al (2020)	Moderate	Low	Low	Low	Low	Low	Low	Low
Woods et al (2020)	Moderate	Moderate	Low	Low	Low	Serious	Moderate	Serious
Zhang et al (2020)	Serious	Low	Low	Low	Low	Serious	Moderate	Serious
Zhou et al (2021)	Moderate	Moderate	Low	Moderate	Low	Critical	Serious	Critical

2.3 MLR								
**Study**	**D1**	**D2**	**D3**	**D4**	**D5**	**D6**	**D7**	**Overall bias**
Afzal et al (2019)	Serious	Low	Moderate	Low	Moderate	Low	Low	Serious
Baucknehtet al (2021)	Moderate	Low	Low	Low	Serious	Moderate	Critical	Critical
Bilen et al (2019)	Moderate	Moderate	Moderate	Low	Low	Low	Moderate	Moderate
Booka et al (2022)	Serious	Low	Low	Moderate	Low	Moderate	Low	Serious
Bronte et al (2022)	Moderate	Low	Low	Low	Low	Serious	Moderate	Serious
Cao et al (2023)	Moderate	Low	Low	Low	Low	Low	Low	Moderate
Chen et al (2021)	Moderate	Low	Low	Low	Low	Low	Low	Moderate
Chen et al (2022)	Moderate	Moderate	Low	Low	Low	Low	Low	Moderate
Cheng et al (2023)	Serious	Serious	Low	Moderate	Moderate	Moderate	Serious	Serious
Da et al (2023)	Moderate	Moderate	Serious	Low	Low	Low	Low	Moderate
Deng et al (2022)	Moderate	Moderate	Low	Low	Low	Moderate	Moderate	Moderate
Dionese et al (2023)	Moderate	Low	Low	Moderate	Low	Serious	Serious	Serious
Failing et al (2017)	Moderate	Low	Low	Low	Low	Low	Serious	Moderate
Fan et al (2021)	Moderate	Low	Low	Low	Low	Low	Low	Low
Goldschmidt et al (2023)	Low	Low	Moderate	Low	Low	Low	Low	Low
Hamai et al 2023	Moderate	Low	Low	Low	Low	Moderate	Moderate	Moderate
Hayano et al (2023)	Moderate	Moderate	Low	Low	Low	Moderate	Low	Moderate
Hou et al (2023)	Moderate	Serious	Moderate	Low	Low	Low	Serious	Serious
Huang et al (2022)	Serious	Moderate	Low	Low	Moderate	Moderate	Critical	Critical
Inoue et al (2022)	Moderate	Low	Low	Low	Low	Moderate	Low	Moderate
Ishihara et al (2019)	Moderate	Moderate	Low	Low	Low	Low	Low	Low
Jeon et al (2022)	Moderate	Moderate	Low	Low	Low	Low	Serious	Serious
Jiang et al (2021)	Moderate	Low	Low	Low	Low	Low	Serious	Moderate
Katayama et al (2020)	Moderate	Low	Low	Low	Low	Moderate	Moderate	Moderate
Kikuchi et al (2022)	Moderate	Moderate	Low	Low	Low	Low	Serious	Serious
Krebs et al (2021)	Moderate	Low	Low	Moderate	Low	Serious	Serious	Serious
Li et al (2022)	Moderate	Low	Low	Low	Low	Low	Moderate	Low
Liao et al (2021)	Moderate	Moderate	Low	Low	Low	Low	Low	Moderate
Liu et al (2023)	Serious	Serious	Moderate	Low	Low	Serious	Moderate	Serious
Ma et al (2022)	Moderate	Low	Moderate	Low	Low	Low	Moderate	Moderate
Mei et al (2021)	Moderate	Low	Low	Low	Low	Low	Serious	Moderate
Michailidou et al 2021)	Moderate	Moderate	Low	Low	Low	Moderate	Moderate	Moderate
Niwa et al (2020)	Moderate	Low	Low	Low	Low	Moderate	Low	Moderate
Ouyang et al (2023)	Moderate	Moderate	Moderate	Moderate	Low	Moderate	Serious	Serious
Pang et al (2023)	Moderate	Low	Moderate	Low	Moderate	Moderate	Moderate	Moderate
Prelaj et al (2020)	Low	Low	Moderate	Low	Low	Low	Low	Moderate
Qi et al (2021)	Moderate	Moderate	Low	Low	Low	Low	Moderate	Moderate
Qi et al (2023)	Moderate	Moderate	Low	Low	Low	Low	Low	Moderate
Qiu et al (2023)	Low	Moderate	Low	Low	Low	Low	Low	Low
Pour et al (2021)	Serious	Serious	Low	Low	Low	Low	Moderate	Serious
Rebuzzi et al (2021)	Serious	Low	Low	Low	Low	Moderate	Moderate	Serious
Rijnders et al (2022)	Moderate	Low	Low	Moderate	Low	Moderate	Serious	Serious

Rossi et al (2020)	Moderate	Low	Low	Low	Low	Low	Low	Moderate
Sakai et al (2023)	Moderate	Moderate	Low	Low	Low	Low	Low	Moderate
Sanchez-Gastaldo et al (2021)	Moderate	Moderate	Low	Low	Low	Low	Low	Moderate
Sekine et al (2018)	Moderate	Low	Low	Moderate	Moderate	Moderate	Low	Moderate
Shao et al (2021)	Moderate	Moderate	Low	Low	Low	Low	Low	Moderate
Soyano et al (2018)	Moderate	Moderate	Moderate	Low	Moderate	Low	Low	Moderate
Starzer et al (2021)	Moderate	Low	Low	Moderate	Moderate	Moderate	Serious	Serious
Takada et al (2020)	Moderate	Moderate	Low	Low	Low	Serious	Moderate	Serious
Tokumaru et al (2021)	Moderate	Low	Low	Low	Low	Moderate	Low	Moderate
Varayathu et al (2021)	Serious	Low	Low	Low	Low	Moderate	Low	Moderate
Wang, X et al (2023)	Low	Low	Low	Low	Low	Serious	Moderate	Serious
Wanh et al (2022)	Moderate	Moderate	Low	Moderate	Low	Low	Low	Moderate
Wen et al (2022)	Serious	Moderate	Low	Moderate	Low	Low	Serious	Serious
Wu et al (2021)	Moderate	Moderate	Moderate	Serious	Moderate	Serious	Serious	Serious
Xiao et al (2020)	Serious	Moderate	Low	Serious	Low	Moderate	Serious	Serious
Xie et al (2023)	Moderate	Low	Moderate	Low	Low	Low	Low	Moderate
Yoshida et al (2022)	Moderate	Low	Low	Low	Low	Low	Low	Low
Yuan et al (2022)	Moderate	Moderate	Low	Low	Low	Moderate	Serious	Moderate
Zhang et al (2022)	Moderate	Low	Low	Low	Low	Moderate	Moderate	Moderate
Zhang et al (2023)	Moderate	Moderate	Serious	Low	Low	Moderate	Serious	Serious
Zheng, F et al (2023)	Serious	Serious	Low	Moderate	Low	Serious	Critical	Critical
Zheng, L et al (2023)	Moderate	Low	Low	Serious	Moderate	Serious	Moderate	Serious
Zhu et al (2022)	Moderate	Low	Low	Low	Low	Moderate	Low	Moderate

2.4 m-MDSCs

Study	D1	D2	D3	D4	D5	D6	D7	Overall bias
B. de Lima et al (2021)	Moderate	Moderate	Low	Low	Low	Moderate	Moderate	Serious
Bronte et al (2022)	Moderate	Low	Low	Low	Low	Low	Low	Low
Gaißler et al (2023)	Moderate	Low	Low	Low	Low	Low	Low	Low
Gebhardt et al (2015)	Moderate	Moderate	Low	Low	Moderate	Moderate	Serious	Serious
Huber et al (2018)	Moderate	Low	Low	Low	Moderate	Serious	Moderate	Serious
Koh et al (2020)	Moderate	Low	Low	Low	Low	Low	Serious	Serious
Limagne et al (2019)	Moderate	Low	Low	Low	Low	Low	Low	Moderate
Martens et al (2016)	Moderate	Low	Low	Low	Low	Serious	Moderate	Serious
Meyer et al (2024)	Moderate	Low	Moderate	Low	Low	Moderate	Low	Moderate
Möller et al (2020)	Moderate	Low	Low	Low	Low	Low	Low	Moderate
Pico de Coaña et al (2017)	Moderate	Moderate	Low	Moderate	Low	Moderate	Moderate	Moderate
Riemann et al (2023)	Moderate	Moderate	Moderate	Low	Low	Moderate	Low	Moderate
Retseck et al (2018)	Moderate	Moderate	Moderate	Low	Low	Moderate	Critical	Critical
Shitara et al (2023)	Low	Low	Low	Low	Low	Moderate	Low	Low
Sun et al (2021)	Moderate	Moderate	Moderate	Low	Low	Moderate	Moderate	Moderate
Tarhini et al (2014)	Moderate	Low	Low	Low	Low	Moderate	Moderate	Moderate
Teshima et al (2022)	Low	Low	Low	Low	Low	Moderate	Serious	Serious
Tomela et al (2023)	Moderate	Low	Low	Low	Low	Moderate	Low	Moderate
Tzeng et al (2018)	Moderate	Moderate	Low	Low	Low	Moderate	Low	Moderate

2.5 irAEs								
**Study**	**D1**	**D2**	**D3**	**D4**	**D5**	**D6**	**D7**	**Overall bias**
Akamatsu et al (2020)	Moderate	Serious	Low	Critical	Low	Critical	Critical	Critical
Chen, Y et al (2023)	Serious	Moderate	Low	Low	Low	Moderate	Moderate	Moderate
Cui et al (2023)	Moderate	Moderate	Low	Low	Low	Low	Low	Moderate
Delivanis et al (2017)	Moderate	Moderate	Low	Low	Low	Moderate	Critical	Critical
Egami et al (2021a)	Moderate	Moderate	Low	Low	Low	Moderate	Moderate	Moderate
Egami et al (2021b)	Moderate	Moderate	Low	Low	Low	Moderate	Low	Moderate
Fan et al (2021)	Moderate	Low	Low	Low	Low	Low	Low	Low
Fujimura et al (2018)	Moderate	Low	Low	Low	Low	Low	Moderate	Moderate
Fujisawa et al (2017)	Moderate	Low	Low	Low	Low	Low	Moderate	Moderate
Garrison et al (2022)	Serious	Serious	Serious	Moderate	Low	Serious	Serious	Serious
Gudd et al (2021)	Moderate	Moderate	Low	Low	Low	Low	Low	Moderate
Guida et al (2021)	Moderate	Low	Low	Low	Low	Serious	Serious	Serious
He et al (2023)	Low	Low	Low	Moderate	Low	Moderate	Serious	Serious
Inoue et al (2022)	Moderate	Low	Low	Low	Low	Moderate	Low	Moderate
Kotwal et al (2020)	Moderate	Low	Moderate	Low	Low	Moderate	Low	Moderate
Lepper et al (2023)	Moderate	Low	Low	Low	Low	Moderate	Moderate	Moderate
Martens et al (2016)	Moderate	Low	Low	Low	Low	Serious	Moderate	Serious
Michailidou et al (2021)	Moderate	Moderate	Low	Low	Low	Moderate	Moderate	Moderate
Möhn et al (2023)	Moderate	Moderate	Low	Low	Low	Low	Moderate	Moderate
Nahar et al (2022)	Moderate	Moderate	Low	Low	Low	Low	Low	Moderate
Nuñez et al (2023)	Low	Low	Low	Low	Low	Low	Low	Low
Oyanagi et al (2021)	Moderate	Low	Low	Low	Low	Moderate	Moderate	Moderate
Park et al (2023)	Low	Low	Low	Low	Low	Low	Moderate	Low
Rose et al (2020)	Moderate	Low	Low	Low	Low	Moderate	Low	Moderate
Sekine et al (2018)	Moderate	Low	Low	Moderate	Moderate	Moderate	Low	Moderate
Sørensen et al (2022)	Moderate	Moderate	Low	Low	Low	Low	Low	Moderate
Soyano et al (2018)	Moderate	Moderate	Moderate	Low	Moderate	Low	Low	Moderate
Suresh et al (2019)	Moderate	Moderate	Low	Low	Low	Moderate	Low	Moderate
Tang et al (2023)	Low	Moderate	Low	Low	Moderate	Moderate	Low	Moderate
Wölffer et al (2022)	Moderate	Low	Low	Low	Low	Moderate	Moderate	Moderate
Ye el al (2021)	Moderate	Moderate	Low	Critical	Low	Moderate	Critical	Critical
Zamora et al (2021)	Moderate	Low	Low	Low	Low	Moderate	Low	Moderate

2.6 GRADE certainty criteria

GRADE certainty criteria	Risk of Bias Summary	AMC	MLR	Monocyte markers	m-MDSCs	irAEs
		**Parameters of Interest**				
**Risk of Bias:** How likely the study design and conduct introduced bias that could skew the results?	Low	19%	9%	7%	15%	9%
	Moderate	35%	52%	61%	50%	69%
	Serious	38%	34%	17%	30%	13%
	Critical	8%	5%	15%	5%	9%
**Study limitations**		Serious limitations	No serious limitations	No serious limitations	No serious limitations	No serious limitations
**Inconsistency:** How much the findings vary across different studies included in the review.		PFS - low Inconsistency<br>OS - high Inconsistency	PFS - low Inconsistency<br>PFS - low Inconsistency	Moderate Inconsistency	Low % of m-MDSCs Inconsistency<br>N/A for other parameters	Moderate Inconsistency
**Indirectness:** How relevant the studies are to the specific question being asked in the review.		Low	Low	Low	Low	Moderate
**Imprecision:** Whether the studies have enough data to provide a reliable estimate of the effect.		Low	Low	Moderate	Moderate	Low
**Publication Bias:** The possibility that studies with negative or null findings were less likely to be published.		N/A as Bayesian meta-analysis was conducted	N/A as Bayesian meta-analysis was conducted	Moderate	Moderate	Moderate

3. Included articles and extracted data

Study	Disease /Primary tumor	N (patients)	N (Healthy controls/ controls)	Study Type	Given ICI	Method
**Adamo et al (2023)**	NSCLC	34	NI	Retrospective	Pembrolizumab<br>Nivolumab<br>Atezolizumab	Flow cytometry, Immunoassay
**Afzal et al (2019)**	Melanoma	120	NI	Retrospective	Ipilimumab<br>Nivolumab<br>Pembrolizumab<br>Ipilimumab plus Nivolumab	Clinical blood count
**Akamatsu et al (2020)**	NSCLC	106	NI	Retrospective	Nivolumab<br>Pembrolizumab<br>Atezolizumab	Multiplex bead immunoassays
**Ando et al (2021)**	NSCLC<br>Gastric cancer<br>Melanoma<br>Parotid cancer<br>Bladder cancer	32	2	Observational	Nivolumab<br>Pembrolizumab	Flow cytometry
**B. De Lima et al (2021)**	Pan-cancer	33	NI	Prospective	Anti-PD-1 plus anti-LAG-3<br>anti-PD-1 plus anti-CTLA-4<br>anti-PD-1<br>anti-PD-L1	Flow cytometry
**Bai R et al (2021)**	Lung cancer<br>Melanoma<br>Esophageal cancer<br>Liver cancer<br>Urothelial cancer<br>Gastric cancer<br>and other types	103	NI	Retrospective	Pembrolizumab<br>Nivolumab<br>Toripalimab<br>Sintilimab<br>Tislelizumab<br>Camrelizumab<br>Atezolizumab)<br>Nivolumab plus Ipilimumab.	Clinical blood count/ Flow cytometry
**Bai X et al (2021)**	Melanoma	89	NI	Phase II	Anti-PD-1	Clinical blood count
**Baucknehet et al (2021)**	NSCLC	45	NI	Translational research trial	Nivolumab	Clinical blood count
**Bilen et al (2019)**	Melanoma<br>Gastrointestinal cancers<br>and lung or head and neck cancers	79 for OS<br>61 for PFS	NI	Prospective	Ipilimumab<br>Nivolumab<br>Pembrolizumab<br>Atezolizumab<br>Avelumab<br>Durvalumab	Clinical blood count
**Booka et al (2022)**	Esophageal squamous cell carcinoma<br>Gastric/gastroesophageal adenocarcinoma	61	NI	Retrospective	Nivolumab or pembrolizumab	Clinical blood count
**Bronte et al (2022)**	NSCLC	22	NI	Prospective	Atezolizumab<br>pembrolizumab<br>Nivolumab	Flow cytometry
**Cao et al (2023)**	Nasopharyngeal carcinoma	184 Training<br>100 Validation	NI		Camrelizumab<br>Toripalimab<br>Sintilimab<br>Nivolumab<br>Pembrolizumab	Clinical blood count
**Chen, X et al (2022)**	Lung cancer	216	NI	Retrospective	Pembrolizumab<br>Tislelizumab<br>Others	Clinical blood count
**Chen et al (2021)**	Gastric cancer	139	NI	Prospective	Anti-PD-1 plus anti-PD-L1	Clinical blood count
**Chen, Y et al (2023)**	NSCLC	269	NI	Retrospective	Pembrolizumab	Clinical blood count
**Cheng, L et al (2023)**	Lung cancer	77	NI	Prospective	Camrelizumab	Clinical blood count
**Chasseuil et al (2018)**	Melanoma	87	NI	Prospective	Nivolumab	Clinical blood count
**Cui et al (2023)**	Checkpoint inhibitor-related pneumonitis	13 (7 CIP)	6	Retrospective	Not indicated	BAL
**Comin-Anduix et al (2010**	Melanoma	27	3	Prospective	Tremelimumab	Flow cytometry

**Da et al (2023)**	Esophageal squamous cell carcinoma	162	NI	Retrospective	Camrelizumab<br>Sintilimab<br>Toripalimab	Clinical blood count
**De Coaña et al (2020)**	Melanoma	36	NI	Prospective	Pembrolizumab<br>Nivolumab	Clinical blood count/ Flow cytometry
**De Coaña et al (2017)**	Melanoma	43	NI	Prospective	Ipilimumab	Flow cytometry
**Delivanis et al (2017)**	Melanoma<br>NSCLC	93<br>7 blood tests	45 healthy/<br>9 thyroiditis	Retrospective	Pembrolizumab	Flow cytometry
**Deng et al (2022)**	NSCLC	115	NI	Retrospective	Camrelizumab<br>Nivolumab<br>PembrolizumabS intilimab<br>Tislelizumab<br>Toripalimab<br>Unknow	Clinical blood count
**Dionese et al (2023)**	Urothelial cancer	72	NI	Retrospective	Avelumab<br>Pembrolizumab	Clinical blood count
**Egami et al (2021)a**	NSCLC	Out of 171	NI	Retrospective multicenter observational	Nivolumab	Clinical blood count
**Egami et al (2021)b**	NSCLC	92 (45 irAEs)	NI	Retrospective multicenter observational	Pembrolizumab	Clinical blood count
**Ende et al (2023)**	esophageal adenocarcinoma	24	NI	Prospective	Atezolizumab	Flow cytometry
**Failing et al (2017)**	Melanoma	133	NI	Retrospective	Pembrolizumab	Clinical blood count
**Fan et al (2021)**	Gastric<br>Colorectal cancer	111	NI	Retrospective	Anti-PD-1	Clinical blood count
**Fujimura et al (Et al (2018)**	Melanoma	46	NI	Retrospective	Nivolumab	Flow cytometry
**Fujisawa et al (2017)**	Melanoma	101	NI	Retrospective	Nivolumab	Clinical blood count
**Gaißler et al (2023)**	melanoma	141	NI	Prospective	Pembrolizumab<br>Nivolumab<br>Nivolumab plus Ipilimumab	Flow cytometry
**Garrison et al (2022)**	skin irAEs	4	unclear	Retrospective	Ipilimumab plus Nivolumab<br>anti-PD-1	single-cell 5′ RNA-seq data analysis
**Goldschmidt et al (2023)**	Melanoma, NSCLC<br>Renal cell carcinoma	18,186	NI	Retrospective	Ipilimumab<br>Pembrolizumab<br>Nivolumab<br>Atezolizumab.	Clinical blood count
**Gebhardt et al (2015)**	Melanoma	59	NI	Retrospective	Ipilimumab	Flow cytometry
**Gudd et al (2021)**	Melanoma	22 with/<br>7 without ICI hepatitis	19	Retrospective	Ipilimumab plus Nivolumab	Flow cytometry
**Guida et al (2021)**	Melanoma	148	NI	Retrospective	Ipilimumab<br>anti-PD-1<br>Ipilimumab plus anti-PD-1	Clinical blood count
**Hamai et al 2023**	Esophageal squamous cell carcinoma	59	NI	Retrospective	Nivolumab	Clinical blood count
**Hayano et al (2023)**	Gastric adenocarcinoma	70	NI	Retrospective	Pembrolizumab<br>Nivolumab	Clinical blood count
**He et al (2023)**	PD-1 inhibitors-associated myocarditis	673 (14 myocarditis)	45	Retrospective	Sintilimab<br>Pembrolizumab<br>Tislelizumab<br>Camrelizumab<br>Nivolumab<br>Toripalimab	Clinical blood count
**Hofbauer et al (2022)**	NSCLC<br>Melanoma<br>Head and neck squamous cell carcinoma<br>Other types	32	NI	Prospective	Pembrolizumab<br>Nivolumab	Flow cytometry

**Hong et al (2022)**	Hepatocellular carcinoma	60	NI	Phase II	Pembrolizumab	Single-cell RNA seq.
**Huang et al (2022)**	Hepatitis B Virus-Induced HCC	110	NI	Retrospective	Anti-PD1	Clinical blood count
**Huber et al (2018)**	Melanoma	49	Included	Retrospective	Ipilimumab Nivolumab	Flow cytometry/RNA seq.
**Hou et al (2023)**	Gastric cancer	41	33 healthy controls		Sintilimab Camrelizumab Tislelizumab	Clinical blood count Plasma samples
**Hung et al (2021)**	HCC	16	Included	Prospective	Nivolumab	Flow cytometry
**Inoue et al (2022)**	Esophageal cancer	41	NI	Retrospective	Nivolumab	Clinical blood count
**Ishihara et al (2019)**	Renal cell carcinoma	58	NI	Retrospective	Nivolumab	Clinical blood count
**Jeon et al (2022)**	HCC	48	NI	Prospective	Nivolumab	Flow cytometry
**Jiang et al (2021)**	NSCLC Melanoma Gastric cancer Other digestive system neoplasms	288	NI	Phase ib	Sintilimab	Clinical blood count
**Juliá et al (2019)**	Renal cell carcinoma NSCLC	25	NI	Retrospective	Anti-PD-1	Clinical blood count
**Katayama et al (2020)**	NSCLC	81	NI	Retrospective	Atezolizumab	Clinical blood count
**Keenan et al (2022)**	Biliary tract cancer	9	8	Prospective	Pembrolizumab plus GM-CSF	Multiplex bead immunoassays Single-cell transcriptomics Epitope sequencing
**Khunger et al (2018)**	NSCLC	109	NI	Retrospective	Nivolumab	Clinical blood count
**Kikuchi et al (2022)**	Renal cell carcinoma	27	NI	Retrospective	Nivolumab plus I pilimumab	Clinical blood count
**Koh et al (2020)**	NSCLC	83 - discovery 49 - validation	NI	Prospective	Pembrolizumab Nivolumab	Flow cytometry
**Kotwal et al (2020)**	ICI-induced Thyroiditis	10 Peripheral blood immunop henotypin g was performed on 6 patients,	44	Prospective	Ipilimumab Nivolumab Pembrolizumab Avelumab Durvalumab	Clinical blood count
**Krebs et al (2021)**	Melanoma	45	NI	Prospective open-label	Ipilimumab Nivolumab Pembrolizumab Ipilimumab plus Nivolumab	Clinical blood count
**Krieg et al (2018)**	Melanoma	20	10	Observational	Anti-PD1	Flow cytometry
**Laza-Briviesca et al (2021)**	NSCLC	29	NI	Prospective phase 1	Nivolumab	Cytokine array/ Flow cytometry
**Lepper et al (2023)**	IRAEs (Melanoma)	31	NI	Retrospective	Pembrolizumab Nivolumab Nivolumab plus Ipilimumab Pembrolizumab plus Ipilimumab	Clinical blood count
**Lee et al (2022)**	HCC	23	NI	Retrospective	Nivolumab	Flow cytometry
**Li et al (2022)**	Esophageal squamous cell carcinoma	44	NI	Retrospective follow-up	Camrelizumab nivolumab pembrolizumab sintilimab	Clinical blood count
**Li et al (2022)**	NSCLC	122 discovery 92 validation	NI	Prospective	Anti-PD-1	Clinical blood count
**Liao et al (2021)**	NSCLC	162	NI	Retrospective	Bevacizumab Pembrolizumab	Clinical blood count
**Limagne et al (2019)**	NSCLC	61	Included	Prospective	Nivolumab	Flow cytometry

**Liu et al (2023)**	hepatic carcinoma	167	NI	Retrospective	Anti-PD-1	Clinical blood count
**Lo Russo et al (2023**	NSCLC	65	NI	Prospective	Pembrolizumab	Flow cytometry
**Lu et al (2019)**	Gastrointestinal tract cancer	56 (5 with hyper progressive disease)	NI	Cohort prospective	Anti-PD-1 therapy<br>Anti-PD-L1 therapy<br>Anti-PD-L1 plus anti-CTLA4 therapy	Multiplex bead immunoassays
**Ma et al (2022)**	Esophageal squamous cell carcinoma	81	NI	Retrospective	Pembrolizumab<br>Sintilimab<br>Nivolumab<br>Toripalimab<br>Camrelizumab<br>Tislelizumab	Clinical blood count
**Ma et al (2023)**	NSCLC	14	4	Retrospective	PD-1/PD-L1 + CTLA4	Flow cytometry
**Martens et al (2016)**	Melanoma	105 identification,<br>104 conformation,<br>406 validation	NI	Prospective	Ipilimumab	Flow cytometry
**Mei et al (2021)**	HCC	442	NI	Retrospective	Anti-PD-1 plus tyrosine kinase inhibitors	Clinical blood count
**Menekse et al (2023)**	NSCLC	144	NI		Nivolumab	Clinical blood count
**Meyer et al (2014)**	Melanoma	49	15	Prospective	Ipilimumab	Flow cytometry
**Michailidou et al (2021)**	irAEs	470 (156 irAEs)	NI	Retrospective	Nivolumab<br>Pembrolizumab<br>Cemiplimab<br>Atezolizumab<br>Durvalumab<br>Avelumab<br>Ipilimumab<br>Tremelimumab	Clinical blood count
**Möhn et al (2023)**	irAEs neurotoxicity	110	NI	Retrospective	Atezolizumab<br>Avelumab<br>Cemiplimab<br>Pembrolizumab<br>Nivolumab<br>Ipilimumab<br>Nivolumab plus Ipilimumab	Serum bead-based ELISA
**Möller et al (2020)**	NSCLC	35	NI	Prospective	Pembrolizumab<br>Nivolumab	Flow cytometry
**Möller et al (2022)**	NSCLC	90	NI	Prospective	Pembrolizumab<br>atezolizumab	Flow cytometry
**Nahar et al (2022)**	irAEs colitis	37	9	Retrospective	Pembrolizumab<br>Nivolumab	CyTOF
**Niwa et al (2020)**	Salivary gland Carcinoma	24	NI	Prospective	Nivolumab	Clinical blood count
**Nuñez et al (2023)**	NSCLC Melanoma	144	NI	Prospective	Pembrolizumab<br>Nivolumab<br>Ipilimumab<br>Ipilimumab plus Nivolumab	Flow cytometry
**Nyakas et al (2019)**	Melanoma	69	NI	Phase IV	Ipilimumab	ELISA/ Flow cytometry
**Ohkuma et al (2023)**	NSCLC<br>Gastric cancer<br>Esophageal cancer	44	NI	Prospective	Pembrolizumab<br>Nivolumab	Flow cytometry
**Okuhira et al (2018)**	Melanoma	16	NI	Retrospective	Nivolumab	Clinical blood count
**Olingy et al (2022)**	NSCLC	26	10 matched prior treatment patients	Prospective	Pembrolizumab<br>Nivolumab	Mass cytometry
**Ouyang et al (2023)**	colorectal cancer	110	NI	Retrospective	anti-PD-1/PD-L1	Clinical blood count
**Oyanagi et al (2021)**	NSCLC	63 (50 for irAEs)	NI	Prospective	Nivolumab<br>Pembrolizumab	Multiplex bead immunoassays

**Ozawa et al (2021)**	NSCLC	106	NI	Prospective	Nivolumab<br>Pembrolizumab<br>Atezolizumab	Flow cytometry
**Pang et al (2023)**	Pulmonary lymphoepithelioma-like carcinoma	96	NI	Retrospective	Pembrolizumab<br>Sintilimab<br>Nivolumab<br>Toripalimab<br>Camrelizumab<br>Tislelizumab<br>Durvalumab	Clinical blood count
**Parikh et al (2018)**	NSCLC	32	NI	Retrospective	Pembrolizumab<br>Nivolumab<br>Nivolumab > Pembrolizumab.	Clinical blood count
**Park et al (2023)**	irAEs (NSCLC)	157	NI	Retrospective	Durvalumab	Clinical blood count
**Pedersen et al (2020)**	Melanoma	16	NI	Prospective	Pembrolizumab<br>Ipilimumab plus Nivolumab	Proximity extension assay
**Pettinella et al (2023)**	NSCLC	30	NI	Retrospective	Pembrolizumab<br>Nivolumab<br>Durvalumab<br>Atezolizumab	Flow cytometry
**Pirozyan et al (2020)**	Melanoma	42	NI	Prospective	Pembrolizumab<br>Nivolumab	Flow cytometry
**Prelaj et al (2020)**	NSCLC	154	NI	Retrospective	Nivolumab<br>Pembrolizumab	Clinical blood count
**Pu et al (2021)**	NSCLC	184	NI	Retrospective	Anti-PD-1	Clinical blood count
**Qi et al (2021)**	SCLC	53	NI	Prospective	Atezolizumab	Clinical blood count
**Qi et al (2023)**	Esophageal squamous cell carcinoma	51	NI	Retrospective	(neo-CRT) and Pembrolizumab.	Clinical blood count
**Qiu et al (2023)**	Pancreatic cancer	67	NI	Retrospective	Toripalimab<br>Sintilimab<br>Pembrolizumab	Clinical blood count
**Pour et al (2021)**	Melanoma	8 discovery<br>20 validation	NI	Prospective	Nivolumab	Single-cell RNA seq.
**Rapposelli et al (2021)**	HCC	10 (blood from 4)	NI	Retrospective	Nivolumab	Clinical blood count
**Rebuzzi et al (2021)**	Renal cell carcinoma		NI	Retrospective	Nivolumab	Clinical blood count
**Retseck et al (2018)**	Melanoma	31	NI	Prospective	Neoadjuvant Ipilimumab	Flow cytometry
**Ribas et al (2016)**	Melanoma	53	1	Phase I	Pembrolizumab	Flow cytometry
**Riemann et al (2020)**	NSCLC	35	NI	Prospective	Pembrolizumab	Flow cytometry
**Riemann et al (2023)**	Small Cell Lung Cancer, NSCLC	40	84	Retrospective	Atezolizumab	Flow cytometry
**Rijnders et al (2022)**	Urothelial carcinoma	71	NI	Phase ii	Pembrolizumab	Flow cytometry + clinical tests
**Rochigneux et al (2022)**	NSCLC	27	NI	Retrospective	Pembrolizumab	CyTOF
**Romano et al (2015)**	Melanoma	29	Included	Prospective	Ipilimumab	Flow cytometry
**Rose et al (2020)**	Melanoma<br>Renal cell carcinoma<br>Urothelial cancer<br>NSCLC	288	NI	Retrospective	Nivolumab	Clinical blood count
**Rosner et al (2018)**	Melanoma	209	NI	Retrospective	Nivolumab plus Ipilimumab	Clinical blood count
**Rossi et al (2020)**	NSCLC	65	NI	Retrospective	Nivolumab	Clinical blood count
**Rossi et al (2022)**	Cutaneous melanoma	87	NI	Prospective	Ipilimumab<br>Nivolumab<br>pembrolizumab<br>Ipilimumab plus Nivolumab	Multiplex bead immunoassays
**Sakai et al (2023)**	Head and neck cell carcinomas	102	NI	Retrospective	Pembrolizumab<br>Nivolumab	Clinical blood count
**Sanchez-Gastaldo et al (2021)**	NSCLC	51	NI	Retrospective	Pembrolizumab	Clinical blood count
**Sekine et al (2018)**	NSCLC	87 Identificat	NI	Retrospective	Nivolumab	Clinical blood count

		ion<br>75<br>validation				
**Shao et al (2021)**	Pan-cancer patients	107	NI	Prospective	ATEZOLIZUMAB<br>CS1001<br>HX008<br>LP002<br>SHR1316	Clinical blood count
**Shitara et al (2023)**	Gastric or gastroesophageal junction cancer	137	NI	Prospective	Pembrolizumab	RNA-Sequencing
**Sørensen et al (2022)**	irAE arthritis	28 synovial fluid<br>6 blood	6	Prospective	Pembrolizumab	Clinical blood count
**Soyano et al (2018)**	NSCLC	157	NI	Retrospective	Nivolumab<br>Pembrolizumab	Clinical blood count
**Starzer et al (2021)**	Sarcomas	35	NI	Retrospective	Pembrolizumab<br>Nivolumab	Clinical blood count
**Sun et al (2021)**	Melanoma	128	NI	Prospective	Ipilimumab plus Nivolumab<br>Nivolumab<br>Pembrolizumab	Flow cytometry
**Suresh et al (2019)**	Checkpoint inhibitor pneumonitis (CIP)<br>NSCLC<br>Melanoma	12 with/<br>6 without pneumonitis	NI	Prospective	Nivolumab<br>Pembrolizumab<br>Durvalumab	Flow cytometry
**Takada et al (2020)**	NSCLC	226	NI	Retrospective	Nivolumab<br>Pembrolizumab	Clinical blood count
**Tang et al (2023)**	ICI-associated myocarditis	81	NI	Retrospective	SI-B003<br>Sintilimab<br>Nivolumab<br>Durvalumab	Clinical blood count
**Tanizaki et al (2018)**	NSCLC	134	NI	Prospective	Nivolumab	Clinical blood count
**Tarhini et al (2014)**	Melanoma	35 (33 analysed)	NI	Prospective	Ipilimumab	Flow cytometry
**Teshima et al (2022)**	Urothelial carcinoma	31	NI	Prospective	Pembrolizumab	Flow cytometry
**Tokumaru et al (2021)**	Gastric Cancer	71	NI	Retrospective	Nivolumab	Clinical blood count
**Tomela et al (2023)**	Melanoma	46	9		Pembrolizumab<br>Nivolumab	Flow cytometry
**Troiani et al (2020)**	Melanoma	22	27	Prospective	Anti-PD1	Flow cytometry
**Tzeng et al (2018)**	Urothelial carcinoma	41	NI	Prospective	Atezolizumab<br>Avelumab<br>Pembrolizumab	Flow cytometry
**Varayathu et al (2021)**	NSCLC<br>Melanoma<br>Head and neck cancer<br>Renal cell carcinoma<br>Gastrointestinal malignancies<br>Dual malignancy<br>Breast cancer	61	NI	Retrospective	Pembrolizumab<br>Nivolumab	Clinical blood count
**Wang et al (2019)**	Esophageal squamous cell carcinoma	43	NI	Retrospective	Camrelizumab	Clinical blood count
**Wang et al (2022)**	Lung cancer	125	NI	Prospective	Nivolumab<br>pembrolizumab<br>atezolizumab	Clinical blood count
**Wang, X et al (2023)**	NSCLC	159	85 Validation cohort	Retrospective	Anti-PD-1/PD-L1	Flow cytometry
**Wen et al (2022)**	NSCLC	90	NI	Retrospective	Anti-PD-1	Clinical blood count
**Wölffer et al (2022)**	Melanoma	95	NI	Prospective	Anti-PD-1<br>anti-PD-1 anti CTLA-4	Clinical blood count
**Woods et al (2020)**	Melanoma	37	NI	Prospective	Nivolumab > Ipilimumab	Flow cytometry

					Ipilimumab > Nivolumab	
**Wu et al (2021)**	Esophageal squamous cell carcinoma	119	818	Retrospective	Camrelizumab Nivolumab pembrolizumab toripalimab sintilimab.	Clinical blood count
**Xiao et al (2020)**	Melanoma Renal cell carcinoma Liver cancer NSCLC	121	NI	Retrospective	Anti-PD-1	Clinical blood count
**Xie et al (2023)**	Small cell lung cancer	83	NI	Retrospective	Anti-PD-1/PD-L1	Clinical blood count
**Ye et al (2021)**	Melanoma	144 and 211 (independent cohorts)	NI	Retrospective	Pembrolizumab Nivolumab Ipilimumab Nivolumab plus Ipilimumab	Poly(A) RNA seq./ Clinical blood count
**Yoshida et al (2022)**	Urothelial carcinoma with liver metastasis	899	NI	Prospective	Atezolizumab pembrolizumab	Clinical blood count
**Yuan et al (2022)**	Gastric cancer	80 discovery 357 validation	NI	Retrospective	Anti-PD-1/PD-L1	Clinical blood count
**Zamora et al (2021)**	NSCLC	87	26	Prospective	Pembrolizumab Nivolumab Atezolizumab Avelumab	Flow cytometry
**Zhang, T et al (2020)**	Urothelial cancer		NI	Randomised phase ii	Acalabrutinib plus Pembrolizumab	Flow cytometry
**Zhang, Xet al (2022)**	Esophageal squamous cell carcinoma	64	NI	Retrospective	Anti-PD-1 antibody (Camrelizumab)	Clinical blood count
**Zhang, Z et al (2023)**	Biliary tract cancer	129	NI	Retrospective	not indicated	Clinical blood count
**Zheng, F et al (2023)**	Not indicated	435	NI	Retrospective	PD-1 or PD-L1	Clinical blood count
**Zheng, L et al (2023)**	NSCLC	139	NI	Retrospective	Tislelizumab Sintilimab Pembrolizumab	Clinical blood count
**Zhou et al (2021)**	Head and neck squamous cell carcinomas NSCLC	104	NI	Prospective	Nivolumab Pembrolizumab Atezolizumab Durvalumab Avelumab	Flow cytometry
**Zhu et al (2022)**	HCC	33	NI	Prospective	Nivolumab	Clinical blood count

NI: not included, NSCLC: non-small-cell lung cancer, HCC: Hepatocellular carcinoma

4. HR PFS and OS for absolute monocyte count (AMC)

	Observed HR PFS for AMC			Estimated effect HR PFS for AMC		
**Study**	**HR**	**95% CI Min**	**95% CI Max**	**HR**	**95% CI Min**	**95% CI Max**
**Chasseuil et al (2018)**	6.33	2.40	16.69	2.98	1.40	7.10
**Chen, X et al (2022)**	1.58	1.08	2.33	1.54	1.08	2.21
**Bronte et al (2022)**	2.74	1.49	5.02	2.22	1.32	3.90
**Menekse et al (2023)**	0.54	0.44	0.89	0.61	0.42	0.89
**Prelaj et al (2020)**	2.24	1.45	3.44	2.04	1.38	3.07
**Bai, X et al (2021)**	2.01	0.53	7.61	1.56	0.69	3.69
**Soyano et al (2018)**	1.71	1.06	2.75	1.63	1.07	2.51
**Yuan et al (2022)**	1.50	1.10	2.00	1.48	1.12	1.97
**Ishihara et al (2019)**	1.14	0.49	2.35	1.22	0.64	2.22
**Li et al (2022)**	0.99	0.51	1.92	1.09	0.61	1.92
**Afzal et al (2019)**	0.96	0.34	2.79	1.15	0.54	2.42
**Tanizaki et al (2018)**	0.64	0.17	1.88	1.00	0.42	2.15
**Qi et al (2023)**	0.50	0.17	1.48	0.86	0.36	1.79
**Average**	1.34	1.17	1.56	1.35	0.92	1.92
	**Observed HR OS for AMC**			**Estimated effect HR OS for AMC**		
**Study**	**HR**	**95% CI Min**	**95% CI Max**	**HR**	**95% CI Min**	**95% CI Max**
**Afzal et al (2019)**	3.15	0.29	32.7	1.94	0.39	10.56
**Bai, R et al (2021)**	2.26	0.9	5.7	2.08	0.89	5.00
**Bai, X et al (2021)**	3.62	0.64	20.34	2.47	0.62	9.71
**Bronte et al (2022)**	2.19	1.24	3.86	2.12	1.23	3.64
**Chasseuil et al (2018)**	4.31	1.46	12.74	3.41	1.25	9.15
**Ishihara et al (2019)**	2.47	0.88	6.11	2.24	0.89	5.57
**Katayama et al (2020)**	1.96	1.07	3.57	1.89	1.04	3.37
**Li et al (2022)**	0.59	0.29	1.2	0.63	0.32	1.25
**Menekse et al (2023)**	0.02	0.00	0.06	0.05	0.01	0.20
**Prelaj et al (2020)**	2.56	1.57	4.1	2.47	1.54	3.96
**Pu et al (2021)**	0.8	0.51	1.24	0.81	0.53	1.26
**Rosner et al (2018)**	5.56	2.88	10.74	4.95	2.60	9.37
**Soyano et al (2018)**	1.71	1.06	2.75	1.69	1.06	2.70
**Tanizaki et al (2018)**	0.83	0.24	2.22	0.91	0.33	2.48
**Wang, X et al (2019)**	0.34	0.07	0.5	0.42	0.16	1.08
**Average**	1.5	1.3	1.8	1.3	0.7	2.3
	**Observed HR OS for AMC**			**Random effect HR OS for AMC**		
**Study**	**HR**	**95% CI Min**	**95% CI Max**	**HR**	**95% CI Min**	**95% CI Max**
**Bai, R et al (2021)**	3.98	1.83	8.65	3.10	1.55	6.52
**Bai, X et al (2021)**	0.83	0.74	7.49	0.96	0.37	2.44
**Chasseuil et al (2018)**	6.31	1.50	26.59	3.01	1.03	10.45
**Li et al (2022)**	0.39	0.16	0.94	0.53	0.23	1.16
**Martens et al (2016)**	2.00	1.13	3.54	1.89	1.09	3.25
**Menekse et al (2023)**	1.04	0.76	1.58	1.05	0.74	1.51
**Michailidou et al (2021)**	2.08	1.21	3.58	1.95	1.17	3.29
**Prelaj et al (2020)**	2.21	1.23	3.99	2.04	1.18	3.57
**Pu et al (2021)**	0.37	0.20	0.68	0.44	0.24	0.78
**Rosner et al (2018)**	2.75	1.30	5.80	2.34	1.17	4.77
**Wang, X. et al (2019)**	0.33	0.13	0.84	0.48	0.20	1.11
**Average**	1.28	1.06	1.55	1.25	0.74	2.08
	**Observed HR PFS for AMC**			**Random effect HR PFS for AMC**		
**Study**	**HR**	**95% CI Min**	**95% CI Max**	**HR**	**95% CI Min**	**95% CI Max**
**Chasseuil et al (2018)**	3.50	1.01	12.10	1.62	1.04	3.14
**Chen, X et al (2022)**	1.74	1.07	2.81	1.56	1.12	2.27
**Li Y et al (2022)**	0.76	0.31	1.83	1.28	0.69	1.93
**Menekse et al (2023)**	1.04	0.76	1.58	1.24	0.86	1.68
**Prelaj et al (2020)**	1.77	1.07	2.93	1.56	1.12	2.31
**Yuan et al (2022)**	1.82	1.22	2.71	1.60	1.19	2.24
**Average**	1.45	1.18	1.78	1.45	1.06	1.97

5. HR PFS, OS and response for monocyte lymphocyte ratio (MLR)

	Observed HR PFS for MLR			Random effect HR PFS for MLR		
**Study**	**HR**	**95% CI Min**	**95% CI Max**	**HR**	**95% CI Min**	**95% CI Max**
**Afzal et al (2019)**	1.04	0.37	2.94	1.24	0.57	2.70
**Bronte et al (2022)**	3.85	1.75	8.33	2.86	1.46	5.59
**Cao et al (2023)**	6.58	4	10.87	5.19	3.23	8.34
**Chen et al (2021)**	1.61	1.1	2.38	1.60	1.11	2.28
**Chen, X et al (2022)**	1.47	0.88	2.44	1.48	0.93	2.38
**Chen, Y et al (2023)**	1.42	1.08	1.89	1.43	1.09	1.87
**Cheng et al (2023)**	1.47	1.05	2.08	1.48	1.07	2.05
**Da et al (2023)**	1.57	1.03	2.4	1.56	1.05	2.34
**Dionese et al (2023)**	0.56	0.32	0.97	0.68	0.41	1.13
**Failing et al (2017)**	1.82	1.09	2.94	1.77	1.12	2.78
**Fan et al (2021)**	2.41	1.51	3.84	2.25	1.45	3.46
**Hayano et al (2023)**	0.54	0.32	0.94	0.66	0.40	1.07
**Hou et al (2023)**	1.85	0.94	3.65	1.76	0.96	3.22
**Huang et al (2022)**	1.25	1.17	1.33	1.25	1.17	1.33
**Inoue 2022**	4.63	2.11	10.99	3.15	1.61	6.29
**Ishihara et al (2019)**	2.85	1.5	5.78	2.42	1.35	4.39
**Jiang et al (2021)**	1.04	0.97	1.79	1.07	0.79	1.45
**Katayama et al (2020)**	2.08	1.27	3.33	1.98	1.28	3.07
**Liao et al (2021)**	2.83	1.56	5.18	2.48	1.43	4.32
**Ma et al (2022)**	2.34	1.25	4.4	2.12	1.23	3.67
**Niwa et al (2020)**	9.09	1.75	50	2.73	1.02	7.64
**Prelaj et al (2020)**	2.17	1.45	3.23	2.09	1.42	3.05
**Qi et al (2023)**	0.82	0.27	2.44	1.11	0.50	2.44
**Qiu et al (2023)**	0.5	0.25	1	0.67	0.36	1.24
**Rebuzzi et al (2021)**	1.23	1	1.54	1.24	1.00	1.53
**Rijnders et al (2022)**	4	3	5	3.81	2.97	4.90
**Sakai et al (2023)**	1.66	1.02	2.69	1.64	1.06	2.55
**Sanchez-Gastaldo et al (2021)**	1.67	0.87	3.23	1.63	0.93	2.91
**Shao et al (2021)**	1.4	0.74	2.65	1.43	0.81	2.51
**Takada et al (2020)**	0.49	0.37	0.66	0.53	0.40	0.70
**Wu et al (2021)**	1.21	0.6	2.45	1.30	0.71	2.32
**Xie et al (2023)**	1.08	0.61	1.92	1.16	0.68	1.95
**Zheng, L et al (2023)**	0.53	0.33	0.84	0.62	0.40	0.95
**Average**	1.34	1.28	1.41	1.53	1.20	1.92
	**Observed HR OS for MLR**			**Random effect HR OS for MLR**		
**Study**	**HR**	**95% CI Min**	**95% CI Max**	**HR**	**95% CI Min**	**95% CI Max**
**Afzal et al (2019)**	5.00	2.13	20.00	3.49	1.40	8.65
**Bronte et al (2022)**	6.25	2.44	16.67	4.35	1.92	10.34
**Chen et al (2021)**	1.82	1.20	2.78	1.82	1.21	2.73
**Da et al (2023)**	2.06	1.32	3.21	2.03	1.33	3.10
**Dionese et al (2023)**	0.55	0.29	1.04	0.67	0.36	1.22
**Failing et al (2017)**	3.45	1.69	6.67	3.09	1.66	5.86
**Fan et al (2021)**	2.63	1.44	4.81	2.51	1.42	4.41
**Hamai et al 2023**	1.43	1.10	1.85	1.44	1.12	1.85
**Hou et al (2023)**	1.97	0.83	4.69	1.95	0.93	4.13
**Huang et al (2022)**	1.08	1.02	1.13	1.08	1.03	1.14
**Inoue et al (2022)**	5.84	2.11	20.62	3.81	1.54	9.88
**Ishihara et al (2019)**	5.44	1.83	23.40	3.47	1.33	9.75
**Jeon et al (2022)**	1.31	0.62	2.77	1.41	0.74	2.76
**Jiang et al (2021)**	2.08	1.45	3.03	2.06	1.45	2.95
**Katayama et al (2020)**	3.33	1.82	5.88	3.06	1.80	5.27
**Liao et al (2021)**	8.33	3.07	22.73	5.23	2.27	12.12
**Ma et al (2022)**	4.56	1.74	11.90	3.48	1.55	7.91
**Mei et al (2021)**	0.71	0.54	0.94	0.73	0.56	0.96
**Niwa et al (2020)**	7.14	1.79	25.00	3.97	1.49	11.29

**Ouyang et al (2023)**	0.21	0.10	0.43	0.32	0.16	0.62
**Prelaj et al (2020)**	2.22	1.45	3.45	2.19	1.46	3.31
**Qi et al (2021)**	2.27	1.58	3.26	2.25	1.58	3.17
**Qiu et al (2023)**	0.42	0.20	0.88	0.56	0.28	1.11
**Rebuzzi et al (2021)**	1.45	1.10	1.89	1.46	1.12	1.91
**Rijnders et al (2022)**	3.60	2.00	8.00	3.21	1.71	6.09
**Rossi et al (2020)**	2.63	1.15	5.88	2.43	1.18	5.01
**Sakai et al (2023)**	3.21	1.83	5.63	2.99	1.75	5.08
**Sanchez-Gastaldo et al (2021)**	2.86	1.28	6.25	2.60	1.28	5.35
**Takada et al (2020)**	0.39	0.28	0.53	0.42	0.30	0.58
**Tokumaru et al (2021)**	0.44	0.21	0.93	0.59	0.30	1.17
**Varayathu et al (2021)**	3.27	1.11	9.71	2.68	1.08	6.52
**Wanh et al (2022)**	3.73	1.89	7.35	3.31	1.83	6.02
**Xie et al (2023)**	1.96	1.06	3.57	1.94	1.12	3.40
**Yoshida et al (2022)**	2.11	1.44	3.09	2.10	1.45	3.06
**Yuan et al (2022)**	1.28	1.00	1.69	1.30	1.00	1.68
**Average**	1.18	1.13	1.23	1.85	1.39	2.50
	**Observed HR OS for MLR**			**Random effect HR OS for MLR**		
**Study**	**HR**	**95% CI Min**	**95% CI Max**	**HR**	**95% CI Min**	**95% CI Max**
**Bilen et al (2019)**	2.15	1.25	3.68	2.01	1.23	3.29
**Chen et al.2021**	2.63	1.61	4.17	2.39	1.55	3.75
**Da et al (2023)**	0.85	0.47	1.555	0.98	0.56	1.66
**Fan et al (2021)**	2.18	1.14	4.18	1.98	1.13	3.47
**Ishihara et al (2019)**	3.42	1.06	15.3	2.13	0.90	5.38
**Liao et al (2021)**	5.29	1.91	14.71	2.98	1.39	6.96
**Ma et al (2022)**	4.52	1.68	12.16	2.78	1.33	6.09
**Michailidou et al (2021)**	1.27	1.05	1.54	1.28	1.06	1.54
**Ouyang et al (2023)**	0.27	0.11	0.67	0.55	0.23	1.15
**Prelaj et al.2020**	1.85	1.1	3.03	1.80	1.15	2.84
**Qi et al.2021**	1.28	0.48	3.45	1.38	0.65	2.87
**Rossi et al (2020)**	1.02	0.29	3.7	1.28	0.53	2.97
**Sakai et al (2023)**	2.34	1.13	4.82	2.05	1.12	3.91
**Sanchez-Gastaldo et al (2021)**	2.94	1.32	6.67	2.33	1.19	4.64
**Soyano et al (2018)**	1.05	0.95	1.15	1.05	0.96	1.16
**Takada et al (2020)**	0.62	0.41	0.93	0.70	0.47	1.04
**Tokumaru et al (2021)**	0.46	0.21	0.98	0.68	0.34	1.31
**Wang et al.2022**	2.47	1.16	5.26	2.10	1.10	4.00
**Xie et al (2023)**	1.85	1.01	3.33	1.78	1.05	3.01
**Yoshida et al (2022)**	2.05	1.35	3.12	1.97	1.32	2.93
**Average**	1.19	1.11	1.28	1.52	1.13	2.08
	**Observed HR PFS for MLR**			**Random effect HR PFS for MLR**		
**Study**	**HR**	**95% CI Min**	**95% CI Max**	**HR**	**95% CI Min**	**95% CI Max**
**Bilen et al (2019)**	2.11	1.36	3.3	1.88	1.31	2.77
**Cao et al (2023)**	1.55	1.04	2.3	1.54	1.09	2.18
**Chen et al (2021)**	1.72	1.1	2.63	1.65	1.16	2.37
**Chen, Y et al (2023)**	2.12	1.39	3.25	1.90	1.34	2.76
**Cheng et al (2023)**	1.54	1.09	2.2	1.53	1.11	2.10
**Da et al (2023)**	0.88	0.51	1.56	1.10	0.70	1.68
**Fan et al (2021)**	1.81	1.08	3.06	1.68	1.11	2.61
**Hayano et al (2023)**	0.97	0.47	2.05	1.23	0.73	2.03
**Hou et al (2023)**	1.74	0.63	4.83	1.57	0.89	2.84
**Ishihara et al (2019)**	2.65	1.3	5.86	1.92	1.17	3.36
**Liao et al (2021)**	3.21	1.75	5.85	2.25	1.40	3.77
**Ma et al (2022)**	2.25	1.18	4.3	1.85	1.17	3.08
**Soyano et al (2018)**	1.04	1.02	1.06	1.04	1.02	1.06
**Takada et al (2020)**	0.66	0.45	0.96	0.82	0.56	1.16
**Yuan et al (2022)**	1.52	1.03	2.22	1.51	1.09	2.10
**Average**	1.05	1.03	1.07	1.51	1.06	2.20

Study	Observed OR overall response rate for MLR<br>HR	 95% CI Min	 95% CI Max	Random effect OR overall response rate for MLR<br>HR	 95% CI Min	 95% CI Max
**Bauckneht et al (2021)**	0.95	0.88	1.03	0.94	0.87	1.02
**Rebuzzi et al (2021)**	0.87	0.58	1.30	0.87	0.64	1.18
**Takada et al (2020)**	0.43	0.22	0.84	0.71	0.36	1.05
**Average**	0.93	0.86	1.01	0.86	0.58	1.19
	**Mean MLR responders**	**SD MLR non-responders**		**Mean MLR non-responders**	**SD MLR non-responders**	**Unpaired T-test P value**
**Shao et al (2021)**	0.43	0.03		0.57	0.04	
**Kikuchi et al (2022)**	0.34	0.17		0.52	0.26	
**Li et al (2022)**	0.27	0.10		0.29	0.14	
**Starzer et al (2021)**	0.51	0.25		0.89	0.65	
**Overall**	0.38	0.1		0.57	0.25	0.23

6. Meta-analysis of univariate HR OS and PFS for AMC and MLR

Meta-analysis for absolute monocyte count (A, B) and monocyte /lymphocyte ratio (C, D) as predictors of overall survival (OS) and progression-free survival (PFS) in patients receiving immune checkpoint inhibitors (ICIs).

A: HR PFS for AMC.

B: HR OS for AMC.

C: HR PFS for MLR.

D. HR OS for MLR.

Fixed effect data is presented in black symbols/intervals, and Bayesian estimated effect in grey symbols/intervals.

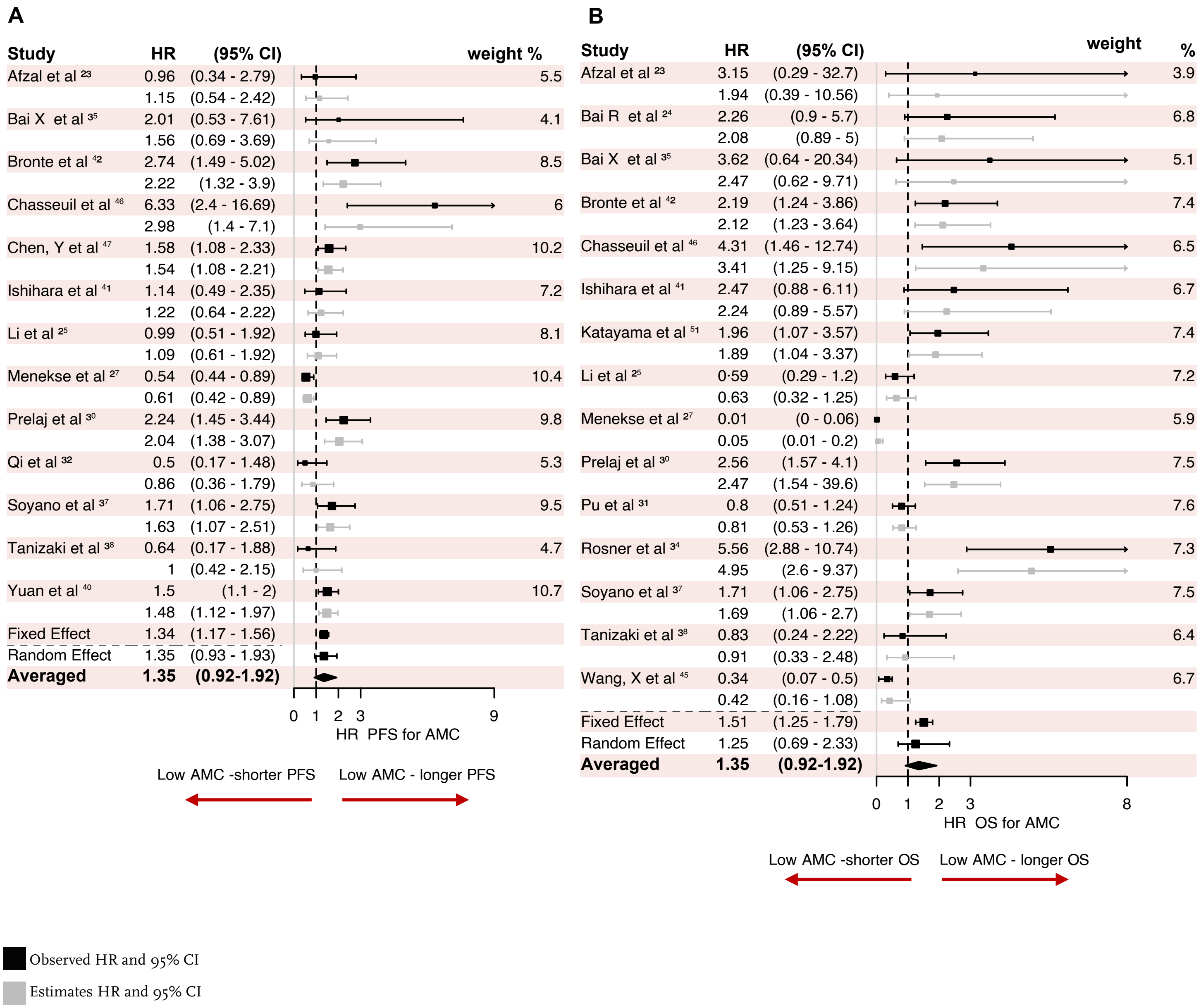

C

Study	HR	(95% CI)	weight %
Afzal et al [23]	1.04	(0.37 - 2.94)	2.1
	1.24	(0.57 - 2.7)	
Bronte et al [42]	3.85	(1.75 - 8.33)	2.6
	2.86	(1.46 - 5.59)	
Cao et al [103]	6.58	(4 - 10.87)	3.2
	5.19	(3.23 - 8.34)	
Chen et al [53]	1.61	(1.1 - 2.38)	3.4
	1.6	(1.11 - 2.28)	
Chen, X et al [47]	1.47	(0.88 - 2.44)	3.2
	1.48	(0.93 - 2.38)	
Chen, Y et al [71]	1.42	(1.08 - 1.89)	3.6
	1.43	(1.09 - 1.87)	
Cheng et al [72]	1.47	(1.05 - 2.08)	3.5
	1.48	(1.07 - 2.05)	
Da et al [73]	1.57	(1.03 - 2.4)	3.3
	1.56	(1.05 - 2.34)	
Dionese et al [74]	0.56	(0.32 - 0.97)	3.1
	0.68	(0.41 - 1.13)	
Failing et al [83]	1.82	(1.09 - 2.94)	3.2
	1.77	(1.12 - 2.78)	
Fan et al [75]	2.41	(1.51 - 3.84)	3.2
	2.25	(1.45 - 3.46)	
Hayano et al [55]	0.54	(0.32 - 0.94)	3.1
	0.66	(0.4 - 1.07)	
Hou et al [76]	1.85	(0.94 - 3.65)	2.8
	1.76	(0.96 - 3.22)	
Huang et al [98]	1.25	(1.17 - 1.33)	3.8
	1.25	(1.17 - 1.33)	
Inoue et al [56]	4.63	(2.11 - 10.99)	2.5
	3.15	(1.61 - 6.29)	
Ishihara et al [41]	2.85	(1.5 - 5.78)	2.8
	2.42	(1.35 - 4.39)	
Jiang et al [84]	1.04	(0.97 - 1.79)	3.5
	1.07	(0.79 - 1.45)	
Katayama et al [51]	2.08	(1.27 - 3.33)	3.2
	1.98	(1.28 - 3.07)	
Liao et al [86]	2.83	(1.56 - 5.18)	3
	2.48	(1.43 - 4.32)	
Ma et al [61]	2.34	(1.25 - 4.4)	2.9
	2.12	(1.23 - 3.67)	
Niwa et al [88]	9.09	(1.75 - 50)	1.2
	2.73	(1.02 - 7.64)	
Prelaj et al [30]	2.17	(1.45 - 3.23)	3.4
	2.09	(1.42 - 3.05)	
Qi et al [89]	0.82	(0.27 - 2.44)	2
	1.11	(0.5 - 2.44)	
Qiu et al [78]	0.5	(0.25 - 1)	2.8
	0.67	(0.36 - 1.24)	
Rebuzzi et al [90]	1.23	(1 - 1.54)	3.6
	1.24	(1 - 1.53)	
Rijnders et al [63]	4	(3 - 5)	3.6
	3.81	(2.97 - 4.9)	
Sakai et al [79]	1.66	(1.02 - 2.69)	3.2
	1.64	(1.06 - 2.55)	
Sanchez-Gastaldo et al [64]	1.67	(0.87 - 3.23)	2.9
	1.63	(0.93 - 2.91)	
Shao et al [36]	1.4	(0.74 - 2.65)	2.9
	1.43	(0.81 - 2.51)	
Takada et al [93]	0.49	(0.37 - 0.66)	3.6
	0.53	(0.4 - 0.7)	
Wu et al [65]	1.21	(0.6 - 2.45)	2.8
	1.3	(0.71 - 2.32)	
Xie et al [80]	1.08	(0.61 - 1.92)	3
	1.16	(0.68 - 1.95)	
Zheng, L et al [68]	0.53	(0.33 - 0.84)	3.2
	0.62	(0.4 - 0.95)	
Fixed Effect	1.34	(1.28 - 1.41)	
Random Effect	1.53	(1.2 - 1.92)	
**Averaged**	**1.53**	**(1.21-1.91)**	

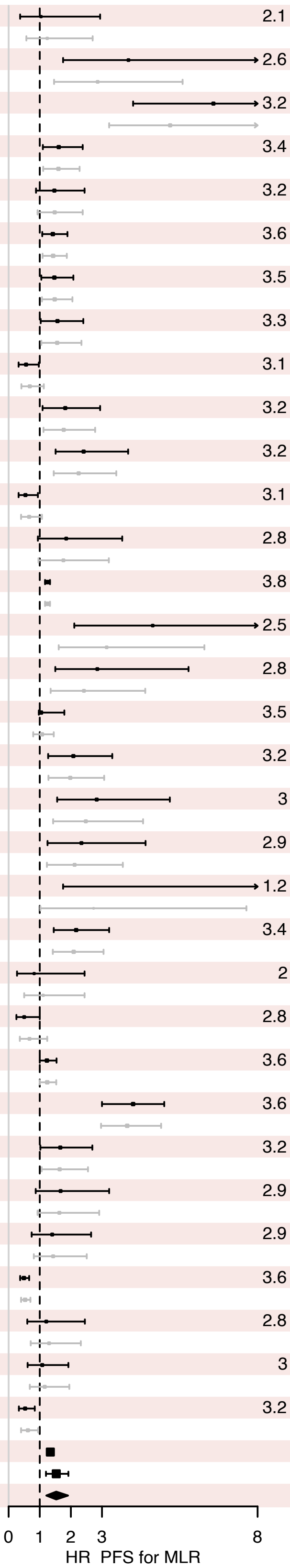

Low MLR -shorter PFS   Low MLR - longer PFS

D

Study	HR	(95% CI)	weight %
Afzal et al [23]	5	(2.13 - 20)	2.2
	3.49	(1.4 - 8.65)	
Bronte et al [42]	6.25	(2.44 - 16.67)	2.4
	4.35	(1.92 - 10.34)	
Chen et al [53]	1.82	(1.2 - 2.78)	3.2
	1.82	(1.21 - 2.73)	
Da et al [73]	2.06	(1.32 - 3.21)	3.2
	2.03	(1.33 - 3.1)	
Dionese et al [74]	0.55	(0.29 - 1.04)	2.9
	0.67	(0.36 - 1.22)	
Failing et al [83]	3.45	(1.69 - 6.67)	2.9
	3.09	(1.66 - 5.86)	
Fan et al [75]	2.63	(1.44 - 4.81)	3
	2.51	(1.42 - 4.41)	
Hamai et al 2023 [104]	1.43	(1.1 - 1.85)	3.4
	1.44	(1.12 - 1.85)	
Hou et al [76]	1.97	(0.83 - 4.69)	2.6
	1.95	(0.93 - 4.13)	
Huang et al [98]	1.08	(1.02 - 1.13)	3.5
	1.08	(1.03 - 1.14)	
Inoue et al [56]	5.84	(2.11 - 20.62)	2.2
	3.81	(1.54 - 9.88)	
Ishihara et al [42]	5.44	(1.83 - 23.4)	2
	3.47	(1.33 - 9.75)	
Jeon et al [57]	1.31	(0.62 - 2.77)	2.8
	1.41	(0.74 - 2.76)	
Jiang et al [84]	2.08	(1.45 - 3.03)	3.3
	2.06	(1.45 - 2.95)	
Katayama et al [51]	3.33	(1.82 - 5.88)	3
	3.06	(1.8 - 5.27)	
Liao et al [86]	8.33	(3.07 - 22.73)	2.4
	5.23	(2.27 - 12.12)	
Ma et al [61]	4.56	(1.74 - 11.9)	2.4
	3.48	(1.55 - 7.91)	
Mei et al [87]	0.71	(0.54 - 0.94)	3.3
	0.73	(0.56 - 0.96)	
Niwa et al [88]	7.14	(1.79 - 25)	1.9
	3.97	(1.49 - 11.29)	
Ouyang et al [77]	0.21	(0.1 - 0.43)	2.8
	0.32	(0.16 - 0.62)	
Prelaj et al [30]	2.22	(1.45 - 3.45)	3.2
	2.19	(1.46 - 3.31)	
Qi et al [114]	2.27	(1.58 - 3.26)	3.3
	2.25	(1.58 - 3.17)	
Qiu et al [78]	0.42	(0.2 - 0.88)	2.8
	0.56	(0.28 - 1.11)	
Rebuzzi et al [90]	1.45	(1.1 - 1.89)	3.4
	1.46	(1.12 - 1.91)	
Rijnders et al [63]	3.6	(2 - 8)	2.8
	3.21	(1.71 - 6.09)	
Rossi et al [91]	2.63	(1.15 - 5.88)	2.7
	2.43	(1.18 - 5.01)	
Sakai et al [79]	3.21	(1.83 - 5.63)	3
	2.99	(1.75 - 5.08)	
Sanchez-Gastaldo et al [64]	2.86	(1.28 - 6.25)	2.7
	2.6	(1.28 - 5.35)	
Takada et al [93]	0.39	(0.28 - 0.53)	3.3
	0.42	(0.3 - 0.58)	
Tokumaru et al [94]	0.44	(0.21 - 0.93)	2.8
	0.59	(0.3 - 1.17)	
Varayathu et al [95]	3.27	(1.11 - 9.71)	2.3
	2.68	(1.08 - 6.52)	
Wang et al [45]	3.73	(1.89 - 7.35)	2.9
	3.31	(1.83 - 6.02)	
Xie et al [80]	1.96	(1.06 - 3.57)	3
	1.94	(1.12 - 3.4)	
Yoshida et al [66]	2.11	(1.44 - 3.09)	3.2
	2.1	(1.45 - 3.06)	
Yuan et al [40]	1.28	(1 - 1.69)	3.4
	1.3	(1 - 1.68)	
Fixed Effect	1.18	(1.13 - 1.23)	
Random Effect	1.85	(1.39 - 2.5)	
**Averaged**	**1.85**	**(1.38-5.5)**	

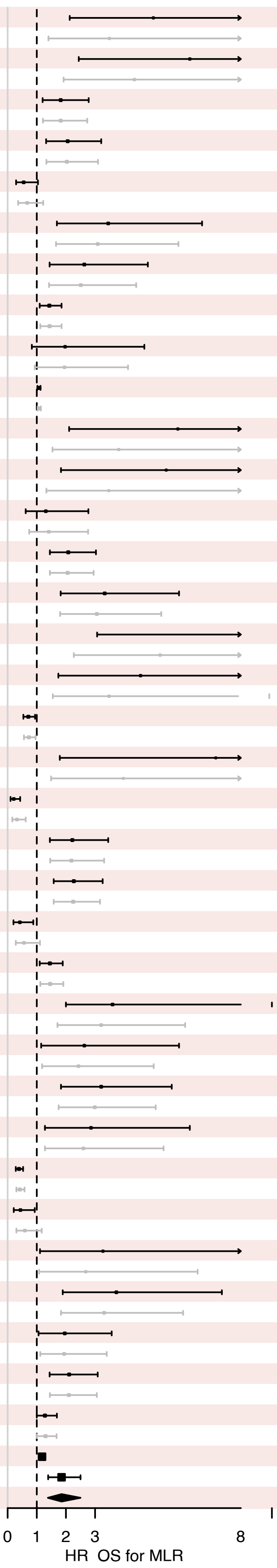

Low MLR -shorter OS   Low MLR - longer OS

■ Observed HR and 95% CI Estimates

■ HR and 95% CI

6.1 Multivariate lnHR OS and PFS for AMC and MLR, stratified by diagnosis and therapeutic target.

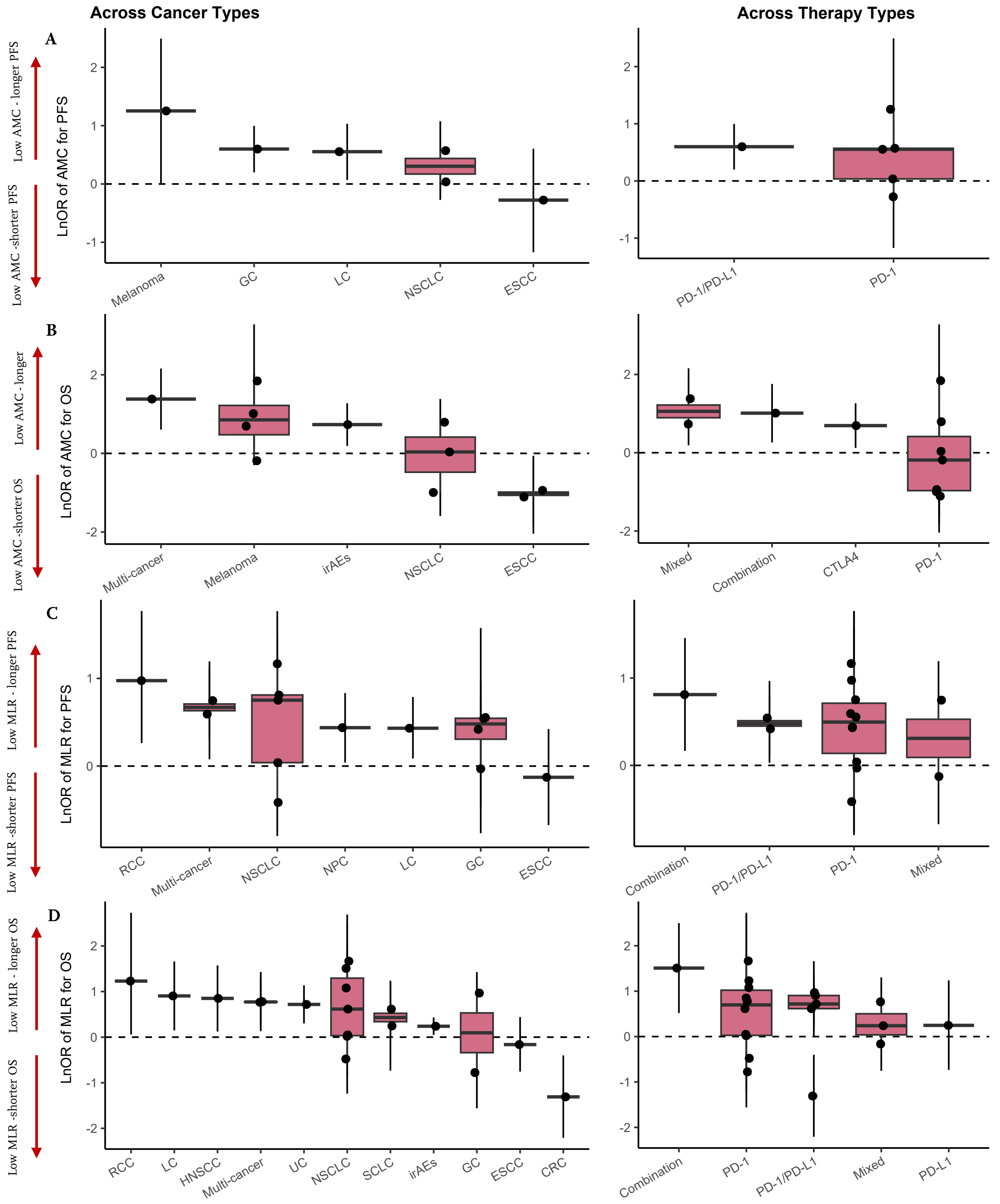

NSCLC: Non-Small Cell Lung Cancer, RCC: Renal Cell Carcinoma, Multi-cancer: study involving various cancer types without specifying a particular one, GC: Gastric Cancer, NPC: Nasopharyngeal Carcinoma,
LC: Lung Cancer, ESCC: Esophageal Squamous Cell Carcinoma, UC: Urothelial Carcinoma, mixed: different types of ICIs in one study pulled together, Combination: combination of PD-1 or PDL1 inhibitors with CTLA-4 inhibitors.
A: HR PFS for AMC. B: HR OS for AMC. C: HR PFS for MLR. D. HR OS for MLR.

7. Cutoff values for MLR and AMC

Study	Cutoff value MLR	Study	Cutoff value AMC
Bilen et al (2019)	0.39	Bai R et al (2021)	620
Ishihara et al (2019)	0.3	Bai X et al (2021)	470
Michailidou et al (2021)	0.73	Ishihara et al (2019)	650
Sanchez-Gastaldo et al (2021)	0.54	Katayama et al (2020	500
Zhu et al (2022)	0.35	Kaushal et al (2018)	700
Rijnders et al (2022)	0.55	Li, Y et al (2022)	315
Yoshida et al (2022)	0.58	Martens et al (2016)	650
Fan et al (2021)	0.31	Michailidou et al (2021)	290
Inoue et al (2022)	0.46	Prelaj et al (2020)	900
Rossi et al (2020)	0.72	Pu et al (2021)	650
Katayama et al (2020)	0.67	Rosner et al (2018)	800
Zhang et al (2022)	0.62	Soyano et al (2018)	630
Failing et al (2017	0.59	Tanizaki et al (2018)	650
Huang et al (2022)	0.56	Wang, X et al (2019)	650
Prelaj et al (2020)	0.56	Chen, X et al (2022)	500
Liao et al (2021)	0.53	Qi et al (2023)	400
Takada et al (2020)	0.47		
Wanh et al (2022)	0.43		
Afzal et al (2019)	0.40		
Tokumaru et al (2021)	0.39		
Rebuzzi et al (2021)	0.38		
Niwa et al (2020)	0.37		
Qi et al (2021)	0.37		
Jiang et al (2021)	0.35		
Chen et al (2021)	0.29		
Mei et al (2021)	0.29		
Xiao et al (2020)	0.24		
Varayathu et al (2021)	0.17		
Da et al (2023)	0.31		
Dionese et al (2023)	0.4		
Hayano et al (2023)	0.29		
Liu et al (2023)	0.28		
Ma et al (2022)	0.40		
Zhang, Z et al (2023)	0.45		
Zheng, F et al (2023)	0.3		
Cao et al (2023)	0.75		
Chen, X et al (2022)	0.23		
Hamai et al 2023	0.29		
Hou et al (2023) (OS)	0.5		
Hou et al (2023) (PFS)	0.32		
Qi et al (2023)	0.30		
Qiu et al (2023)	0.50		
Sakai et al (2023)	0.53		
Xie et al (2023)	0.31		
**25% Percentile**	**0.30**	**25% Percentile**	**477.5**
**Median**	**0.39**	**Median**	**640.0**
**75% Percentile**	**0.54**	**75% Percentile**	**650.0**

8. Monocyte genes as predictors of ICI response.

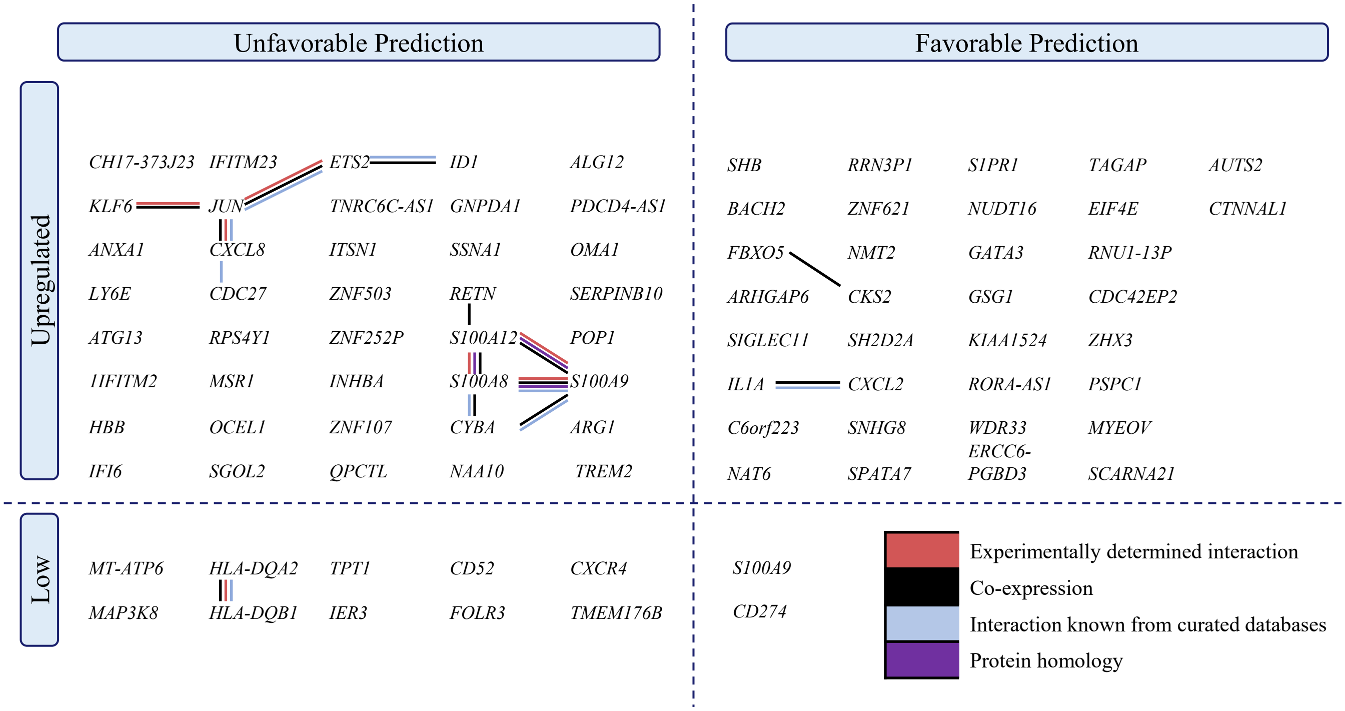

Figure 1. Differentially expressed monocyte-related genes as predictors of response to ICIs. No patterns were identified in the set of genes associated with favourable outcomes. Gene ontology patterns associated with unfavourable prognoses included neutrophil and monocyte migration, neutrophil aggregation, RAGE receptor binding, Toll-like receptor 4 binding, chemotaxis, and the response to hydrogen peroxidase. Among these genes, there were two sets of interconnected genes: transcription factor *JUN* was upregulated along with *CXCL8*, the gene encoding IL18, *KLF6*, a known tumour suppressor, *ETS2* – a protooncogenic factor, and *ID1,* which is strongly associated with immunosuppressive markers of monocytic MDSC.

Additionally, *S100A8, S100A9, and S100A12* were found to be upregulated in non-responders. However, the gene signatures from these studies did not fully overlap.[14,57,102,129] Further gene expression analysis is required to identify specific prognostic patterns.

9. Sensitivity and heterogeneity analyses for random effects models of Bayesian meta-analysis

Studies were excluded one by one from the analysis, and random model Tau and cumulative effects were calculated (see R script for more information)

STUDY	tau_mean	tau_med	tau_2.5%	tau_97.5%	Random effects mean	Random effects mdian	Random effects 2.5%	Random effects 97.5%
**AMC IRAEs**								
**Fujisawa et al (2017)**	0.34	0.27	0.05	1.03	0.95	0.93	0.63	1.66
**Michailidou et al 2021**	0.24	0.20	0.06	0.67	0.91	0.91	0.65	1.29
**Rose et al (2020)**	0.34	0.28	0.08	0.95	1.05	1.03	0.72	1.71
**Soyano et al 2018**	0.33	0.27	0.08	0.95	1.00	0.98	0.68	1.57
**Tang et al (2023)**	0.22	0.16	0.04	0.77	1.16	1.15	0.80	1.71
**AMC OS MV**								
**Bai, R et al (2021)**	0.79	0.75	0.40	1.41	1.14	1.14	0.68	1.92
**Bai, X et al (2021)**	0.88	0.84	0.47	1.54	1.28	1.28	0.74	2.26
**Chasseuil et al (2018)**	0.80	0.76	0.43	1.39	1.15	1.16	0.69	1.92
**Li et al (2022)**	0.79	0.75	0.41	1.41	1.38	1.38	0.81	2.34
**Martens et al (2016)**	0.88	0.84	0.47	1.55	1.20	1.20	0.69	2.11
**Menekse et al (2023)**	0.90	0.86	0.48	1.57	1.27	1.27	0.72	2.27
**Michailidou et al (2021)**	0.88	0.84	0.47	1.54	1.19	1.19	0.69	2.10
**Prelaj et al (2020)**	0.87	0.83	0.46	1.53	1.19	1.19	0.69	2.08
**Pu et al (2021)**	0.72	0.69	0.30	1.34	1.42	1.43	0.85	2.34
**Rosner et al (2018)**	0.85	0.81	0.45	1.50	1.17	1.17	0.68	2.03
**Wang, X et al (2019)**	0.77	0.73	0.39	1.37	1.39	1.39	0.83	2.35
**AMC OS UV**								
**Afzal et al (2019)**	1.27	1.22	0.77	2.01	1.20	1.20	0.64	2.27
**Bai, R et al (2021)**	1.29	1.25	0.78	2.04	1.20	1.19	0.63	2.30
**Bai, X et al (2021)**	1.26	1.22	0.77	2.00	1.18	1.18	0.63	2.24
**Bronte et al (2022)**	1.29	1.25	0.79	2.05	1.19	1.19	0.63	2.30
**Chasseuil et al (2018)**	1.25	1.21	0.75	1.98	1.16	1.16	0.62	2.18
**Ishihara et al (2019)**	1.28	1.24	0.78	2.04	1.19	1.19	0.63	2.28
**Katayama et al (2020)**	1.30	1.25	0.79	2.05	1.20	1.20	0.63	2.32
**Li et al (2022)**	1.27	1.23	0.76	2.03	1.29	1.29	0.68	2.49
**Menekse et al (2023)**	**0.64**	**0.62**	**0.33**	**1.09**	**1.63**	**1.63**	**1.07**	**2.46**
**Prelaj et al (2020)**	1.28	1.24	0.78	2.04	1.18	1.18	0.62	2.27
**Pu et al (2021)**	1.29	1.25	0.78	2.05	1.27	1.27	0.66	2.46
**Rosner et al (2018)**	1.20	1.16	0.70	1.93	1.14	1.14	0.62	2.10
**Soyano et al (2018)**	1.30	1.26	0.79	2.06	1.21	1.21	0.63	2.34
**Tanizaki et al (2018)**	1.29	1.25	0.78	2.05	1.26	1.26	0.66	2.43
**Wang, X et al (2019)**	1.23	1.19	0.73	1.97	1.34	1.33	0.71	2.54
**AMC PFS MV**								
**Chasseuil et al (2018)**	0.21	0.17	0.04	0.61	1.39	1.40	1.01	1.87
**Chen, X et al (2022)**	0.25	0.20	0.04	0.76	1.39	1.39	0.97	1.99
**Li Y et al (2022)**	0.21	0.17	0.04	0.61	1.53	1.53	1.13	2.11
**Menekse et al (2023)**	0.17	0.13	0.04	0.58	1.64	1.65	1.17	2.24
**Prelaj et al (2020)**	0.25	0.20	0.04	0.75	1.39	1.39	0.97	1.98
**Yuan et al (2022)**	0.23	0.18	0.04	0.73	1.36	1.35	0.96	1.94
**AMC PFS UV**								
**Afzal et al (2019)**	0.55	0.52	0.27	0.97	1.37	1.37	0.94	1.98
**Bai, X et al (2021)**	0.54	0.52	0.27	0.96	1.33	1.33	0.91	1.90
**Bronte et al (2022)**	0.51	0.48	0.25	0.92	1.27	1.27	0.89	1.81
**Chasseuil et al (2018)**	0.44	0.42	0.22	0.78	1.25	1.25	0.90	1.70
**Chen, X et al (2022)**	0.57	0.54	0.29	1.00	1.32	1.32	0.90	1.94
**Ishihara et al (2019)**	0.56	0.53	0.28	0.98	1.36	1.36	0.93	1.98
**Li et al (2022)**	0.55	0.52	0.28	0.98	1.38	1.38	0.94	2.00
**Menekse et al (2023)**	0.24	0.20	0.04	0.66	1.57	1.58	1.19	1.99
**Prelaj et al (2020)**	0.53	0.50	0.25	0.95	1.28	1.28	0.89	1.85
**Qi et al (2023)**	0.51	0.48	0.26	0.90	1.42	1.42	1.00	2.02
**Soyano et al (2018)**	0.56	0.53	0.28	0.99	1.32	1.32	0.90	1.92
**Tanizaki et al (2018)**	0.53	0.50	0.27	0.93	1.40	1.40	0.97	2.00

**Yuan et al (2022)**	0.57	0.54	0.29	1.00	1.33	1.33	0.90	1.95
**MLR IRAEs**								
**Chen, Y et al (2023)**	0.99	0.92	0.31	2.13	0.97	0.96	0.46	2.09
**Egami et al (2021)**	1.24	1.14	0.57	2.48	0.88	0.88	0.37	2.10
**Egami et al (2021)**	**0.85**	**0.78**	**0.37**	**1.75**	**0.66**	**0.66**	**0.33**	**1.33**
**Fan et al (2021)**	1.25	1.15	0.57	2.51	0.82	0.82	0.34	1.99
**Inoue et al (2022)**	1.12	1.03	0.51	2.25	0.93	0.93	0.43	2.11
**Michailidou et al (2021)**	1.18	1.08	0.52	2.37	0.92	0.92	0.40	2.13
**Park et al (2023)**	1.01	0.92	0.38	2.15	0.70	0.70	0.32	1.58
**MLR OS MV**								
**Bilen et al (2019)**	0.59	0.58	0.35	0.93	1.49	1.48	1.09	2.04
**Chen, Y et al (2021)**	0.58	0.56	0.33	0.92	1.47	1.46	1.08	2.00
**Da et al (2023)**	0.58	0.56	0.34	0.91	1.56	1.56	1.15	2.14
**Fan et al (2021)**	0.59	0.58	0.35	0.93	1.49	1.49	1.09	2.04
**Ishihara et al (2019)**	0.58	0.56	0.34	0.91	1.48	1.48	1.09	2.01
**Liao et al (2021)**	0.54	0.53	0.31	0.86	1.45	1.45	1.08	1.94
**Ma et al (2022)**	0.55	0.54	0.32	0.88	1.45	1.45	1.08	1.96
**Michailidou et al (2021)**	0.61	0.59	0.36	0.95	1.53	1.53	1.11	2.12
**Ouyang et al (2023)**	0.48	0.47	0.28	0.77	1.62	1.61	1.24	2.14
**Prelaj et al.2020**	0.60	0.58	0.35	0.94	1.50	1.50	1.09	2.06
**Qi et al.2021**	0.59	0.58	0.35	0.93	1.53	1.52	1.12	2.09
**Rossi et al (2020)**	0.59	0.57	0.35	0.92	1.53	1.53	1.13	2.10
**Sakai et al (2023)**	0.59	0.57	0.34	0.93	1.48	1.48	1.09	2.03
**Sanchez-Gastaldo et al (2021)**	0.58	0.56	0.33	0.91	1.47	1.47	1.08	2.00
**Soyano et al (2018)**	0.59	0.58	0.34	0.93	1.56	1.56	1.13	2.14
**Takada et al (2020)**	0.52	0.51	0.27	0.85	1.60	1.60	1.20	2.15
**Tokumaru et al (2021)**	0.53	0.51	0.30	0.84	1.60	1.60	1.20	2.15
**Wang, X et al (2022)**	0.59	0.57	0.34	0.92	1.48	1.48	1.09	2.03
**Xie et al (2023)**	0.60	0.58	0.35	0.94	1.50	1.50	1.09	2.06
**Yoshida et al (2022)**	0.60	0.58	0.35	0.94	1.49	1.49	1.09	2.05
**MLR OS UV**								
**Afzal et al (2019)**	0.77	0.76	0.56	1.04	1.81	1.80	1.36	2.41
**Bronte et al (2022)**	0.76	0.75	0.56	1.02	1.79	1.79	1.35	2.39
**Chen, Y et al (2021)**	0.79	0.78	0.58	1.06	1.85	1.85	1.38	2.49
**Da et al (2023)**	0.79	0.78	0.58	1.06	1.84	1.84	1.38	2.48
**Dionese et al (2023)**	0.75	0.74	0.55	1.02	1.91	1.91	1.45	2.55
**Failing et al (2017)**	0.78	0.77	0.57	1.05	1.81	1.81	1.36	2.43
**Fan et al (2021)**	0.78	0.77	0.58	1.05	1.83	1.83	1.37	2.46
**Hamai et al (2023)**	0.79	0.78	0.58	1.06	1.86	1.86	1.39	2.51
**Hou et al (2023)**	0.78	0.77	0.58	1.06	1.85	1.84	1.38	2.48
**Huang et al (2022)**	0.78	0.77	0.57	1.05	1.88	1.88	1.41	2.53
**Inoue et al (2022)**	0.76	0.75	0.56	1.03	1.80	1.80	1.36	2.40
**Ishihara et al (2019)**	0.77	0.76	0.56	1.03	1.81	1.81	1.36	2.41
**Jeon et al (2022)**	0.78	0.77	0.58	1.05	1.87	1.86	1.40	2.51
**Jiang et al (2021)**	0.79	0.78	0.58	1.06	1.84	1.84	1.38	2.48
**Katayama et al (2020)**	0.78	0.77	0.57	1.05	1.81	1.81	1.36	2.43
**Liao et al (2021)**	0.75	0.74	0.55	1.01	1.78	1.78	1.35	2.36
**Ma et al (2022)**	0.77	0.76	0.56	1.04	1.81	1.80	1.36	2.42
**Mei et al (2021)**	0.76	0.75	0.56	1.03	1.91	1.91	1.44	2.55
**Niwa et al (2020)**	0.76	0.75	0.56	1.02	1.80	1.80	1.36	2.39
**Ouyang et al (2023)**	0.69	0.68	0.50	0.93	1.95	1.95	1.51	2.55
**Prelaj et al (2020)**	0.79	0.78	0.58	1.06	1.84	1.84	1.38	2.47
**Qi et al (2021)**	0.79	0.78	0.58	1.06	1.84	1.83	1.37	2.47
**Qiu et al (2023)**	0.74	0.73	0.54	1.00	1.92	1.92	1.46	2.55
**Rebuzzi et al (2021)**	0.79	0.78	0.58	1.06	1.86	1.86	1.39	2.51
**Rijnders et al (2022)**	0.78	0.76	0.57	1.04	1.81	1.81	1.36	2.43
**Rossi et al (2020)**	0.78	0.77	0.57	1.05	1.83	1.83	1.37	2.46
**Sakai et al (2023)**	0.78	0.77	0.57	1.05	1.82	1.81	1.36	2.44
**Sanchez-Gastaldo et al (2021)**	0.78	0.77	0.57	1.05	1.83	1.82	1.37	2.45
**Takada et al (2020)**	0.71	0.70	0.51	0.97	1.94	1.94	1.48	2.56

**Tokumaru et al (2021)**	0.75	0.73	0.55	1.00	1.92	1.92	1.46	2.55
**Varayathu et al (2021)**	0.78	0.77	0.57	1.05	1.82	1.82	1.37	2.44
**Wang, X et al (2022)**	0.77	0.76	0.57	1.04	1.81	1.81	1.36	2.42
**Xie et al (2023)**	0.79	0.78	0.58	1.06	1.84	1.84	1.38	2.48
**Yoshida et al (2022)**	0.79	0.78	0.58	1.06	1.84	1.84	1.38	2.48
**Yuan et al (2022)**	0.79	0.77	0.58	1.06	1.87	1.87	1.40	2.52
**MLR PFS MV**								
**Bilen et al (2019)**	0.35	0.34	0.18	0.60	1.46	1.46	1.16	1.86
**Cao et al (2023)**	0.37	0.35	0.20	0.62	1.50	1.49	1.18	1.92
**Chen, Y et al (2021)**	0.36	0.35	0.19	0.62	1.49	1.48	1.17	1.90
**Chen, Y et al (2023)**	0.35	0.34	0.18	0.60	1.46	1.45	1.16	1.86
**Cheng et al (2023)**	0.37	0.36	0.20	0.62	1.50	1.50	1.18	1.92
**Da et al (2023)**	0.34	0.33	0.19	0.58	1.55	1.55	1.24	1.96
**Fan et al (2021)**	0.36	0.35	0.19	0.61	1.48	1.48	1.17	1.89
**Hayano et al (2023)**	0.35	0.34	0.19	0.59	1.53	1.53	1.22	1.95
**Hou et al (2023)**	0.36	0.34	0.19	0.60	1.49	1.49	1.19	1.89
**Ishihara et al (2019)**	0.34	0.33	0.18	0.58	1.46	1.46	1.17	1.84
**Liao et al (2021)**	0.32	0.30	0.16	0.54	1.43	1.43	1.16	1.78
**Ma et al (2022)**	0.35	0.34	0.18	0.59	1.47	1.46	1.17	1.86
**Soyano et al (2018)**	0.33	0.32	0.14	0.58	1.57	1.56	1.25	1.97
**Takada et al (2020)**	0.27	0.26	0.14	0.48	1.59	1.58	1.31	1.96
**Yuan et al (2022)**	0.37	0.35	0.20	0.62	1.50	1.50	1.18	1.92
**MLR PFS UV**								
**Afzal et al (2019)**	0.60	0.60	0.44	0.82	1.54	1.54	1.22	1.94
**Bronte et al (2022)**	0.59	0.58	0.43	0.80	1.49	1.49	1.19	1.87
**Cao et al (2023)**	0.54	0.53	0.39	0.74	1.46	1.45	1.18	1.81
**Chen, X et al (2022)**	0.61	0.60	0.45	0.83	1.53	1.53	1.21	1.94
**Chen, Y et al (2021)**	0.61	0.60	0.45	0.83	1.52	1.52	1.20	1.93
**Chen, Y et al (2023)**	0.61	0.60	0.45	0.83	1.53	1.53	1.21	1.94
**Cheng et al (2023)**	0.61	0.60	0.45	0.83	1.53	1.53	1.21	1.94
**Da et al (2023)**	0.61	0.60	0.45	0.83	1.53	1.52	1.21	1.93
**Dionese et al (2023)**	0.58	0.57	0.42	0.79	1.57	1.57	1.26	1.98
**Failing et al (2017)**	0.61	0.60	0.44	0.83	1.52	1.52	1.20	1.92
**Fan et al (2021)**	0.60	0.59	0.44	0.82	1.50	1.50	1.19	1.90
**Hayano et al (2023)**	0.58	0.57	0.42	0.79	1.58	1.58	1.26	1.98
**Hou et al (2023)**	0.61	0.60	0.44	0.83	1.52	1.52	1.20	1.92
**Huang et al (2022)**	0.61	0.60	0.45	0.83	1.54	1.54	1.21	1.95
**Inoue et al (2022)**	0.58	0.58	0.43	0.79	1.48	1.48	1.18	1.86
**Ishihara et al (2019)**	0.60	0.59	0.44	0.81	1.50	1.50	1.19	1.89
**Jiang et al (2021)**	0.61	0.60	0.44	0.82	1.55	1.55	1.22	1.96
**Katayama et al (2020)**	0.61	0.60	0.44	0.83	1.51	1.51	1.20	1.91
**Liao et al (2021)**	0.60	0.59	0.44	0.81	1.50	1.50	1.19	1.89
**Ma et al (2022)**	0.60	0.60	0.44	0.82	1.51	1.51	1.19	1.91
**Niwa et al (2020)**	0.59	0.58	0.43	0.79	1.49	1.49	1.19	1.87
**Prelaj et al (2020)**	0.61	0.60	0.44	0.82	1.51	1.51	1.19	1.91
**Qi et al (2023)**	0.60	0.59	0.44	0.82	1.55	1.54	1.23	1.95
**Qiu et al (2023)**	0.58	0.57	0.42	0.79	1.57	1.57	1.26	1.98
**Rebuzzi et al (2021)**	0.61	0.60	0.45	0.83	1.54	1.54	1.22	1.95
**Rijnders et al (2022)**	0.57	0.56	0.41	0.78	1.47	1.47	1.18	1.85
**Sakai et al (2023)**	0.61	0.60	0.45	0.83	1.52	1.52	1.20	1.93
**Sanchez-Gastaldo et al (2021)**	0.61	0.60	0.44	0.83	1.52	1.52	1.20	1.93
**Shao et al (2021)**	0.61	0.60	0.44	0.83	1.53	1.53	1.21	1.94
**Takada et al (2020)**	0.56	0.55	0.40	0.77	1.59	1.59	1.28	1.98
**Wu et al (2021)**	0.61	0.60	0.44	0.83	1.54	1.54	1.22	1.94
**Xie et al (2023)**	0.61	0.60	0.44	0.82	1.54	1.54	1.22	1.95
**Zheng, L et al (2023)**	0.57	0.56	0.42	0.78	1.58	1.58	1.26	1.98

10. Gating strategies for mMDSCs stratified by the reported effect and diagnosis.

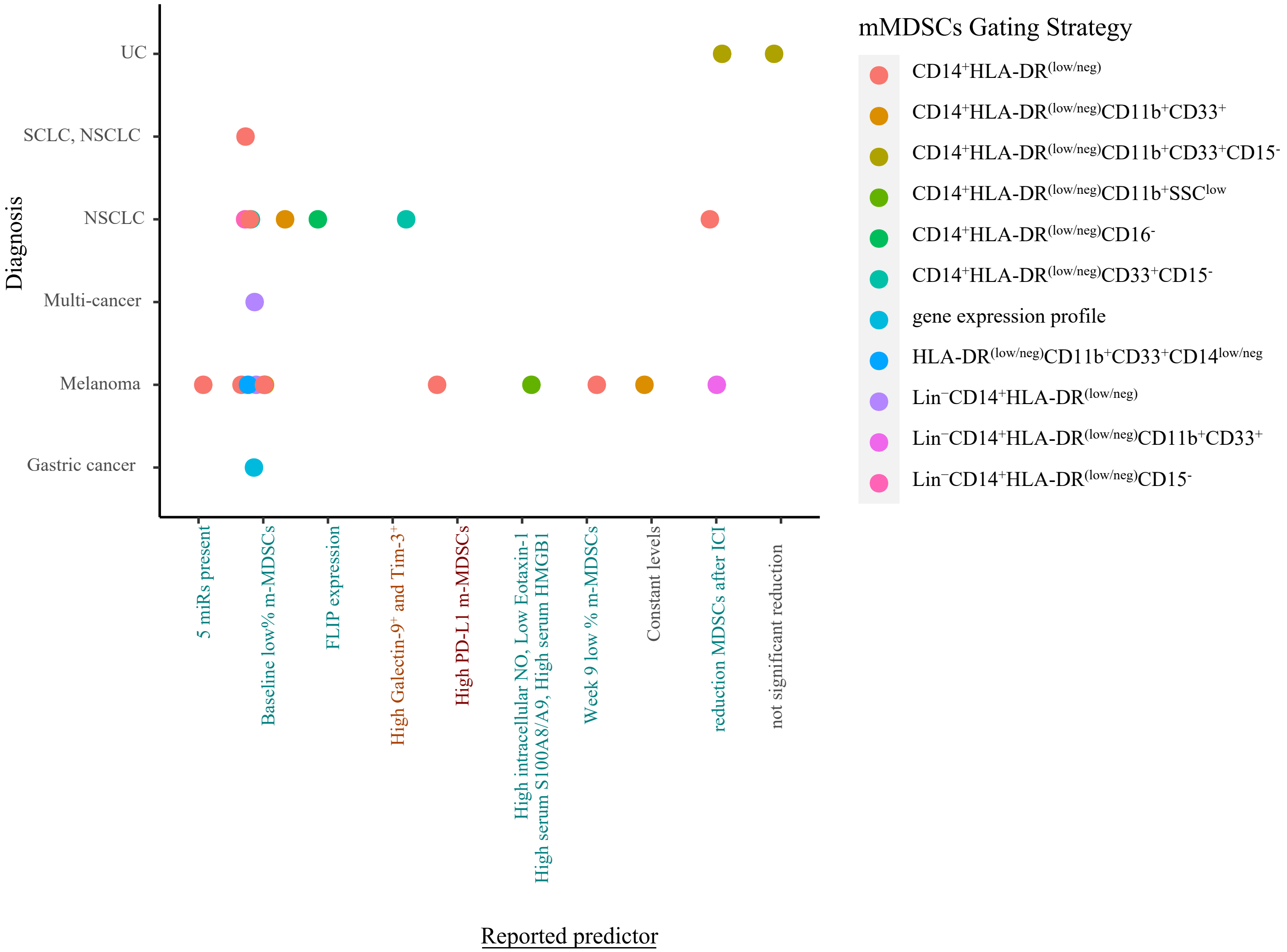

NSCLC: Non-Small Cell Lung Cancer, SCLC: Small Cell Lung Cancer, RCC: Renal Cell Carcinoma, Multi-cancer: study involving various cancer types without specifying a particular one.
cFLIP: cellular FLICE (FADD-like interleukin-1β-converting enzyme)-inhibitory protein,
VISTA: V-domain Ig suppressor of T cell activation,
HMGB1: High mobility group box 1,
S100A8/A9: calcium- and zinc-binding proteins.

11. Studies discussing irAEs and survival outcomes

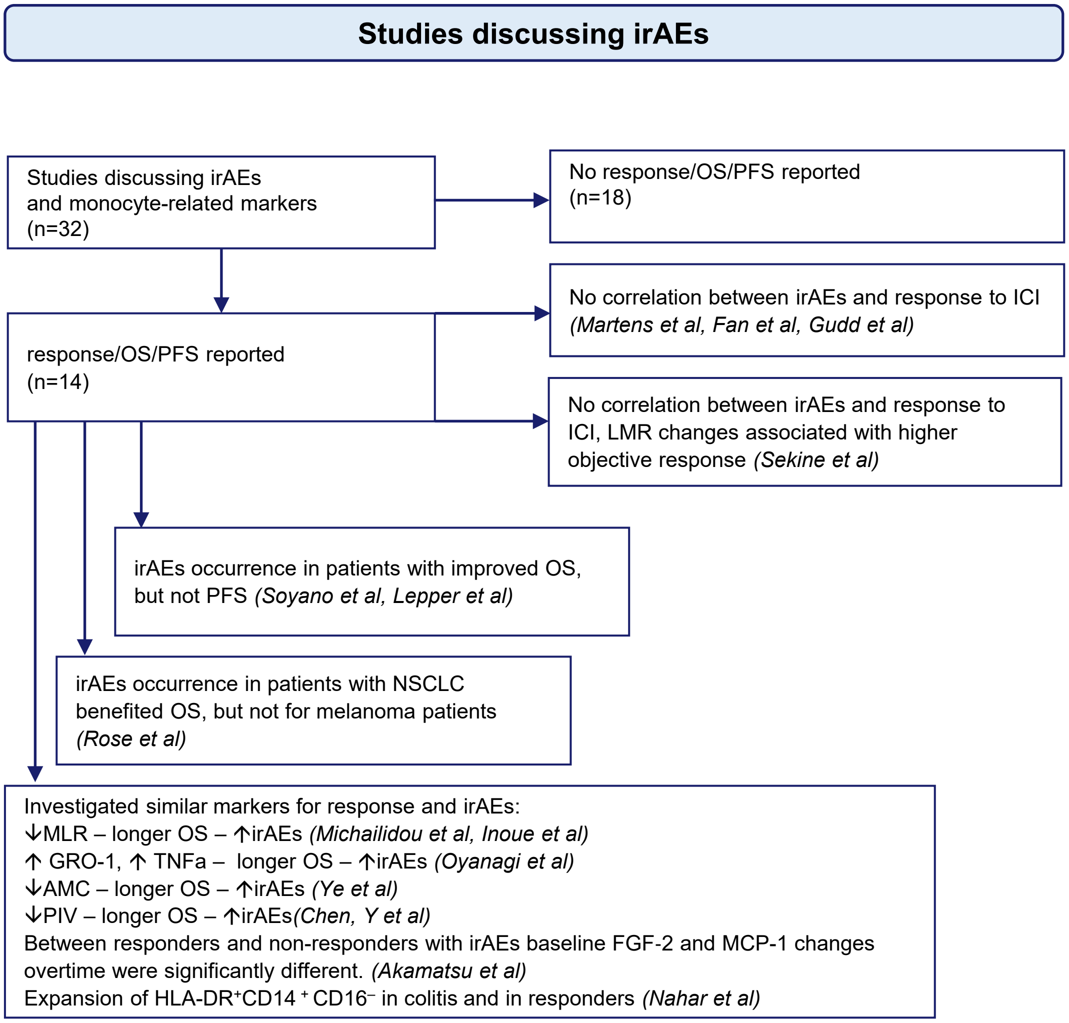

GRO-1: chemokine (C-X-C motif) ligand 1
MCP-1: monocyte chemoattractant protein 1
PIV: paninflammatory value
FGF2: fibroblast growth factor
TNFa: tumor necrosis factor alpha
